# Probing the Electric Double-Layer Capacitance to Understand the Reaction Environment in Conditions of Electrochemical Amination of Acetone

**DOI:** 10.1021/acsami.4c14134

**Published:** 2025-01-02

**Authors:** Yani Guan, Justus Kümper, Simran Kumari, Nick Heiming, Sonja D. Mürtz, Stephan N. Steinmann, Stefan Palkovits, Regina Palkovits, Philippe Sautet

**Affiliations:** † Department of Chemical and Biomolecular Engineering, University of California Los Angeles, Los Angeles, California 90095, United States; ‡ Chair of Heterogeneous Catalysis and Technical Chemistry RWTH Aachen University Worringerweg 2, 52074 Aachen, Germany; § CNRS, Laboratoire de Chimie UMR 5182, ENS de Lyon, 46 allée d’Italie, Lyon F-69342, France; ∥ Institute for Sustainable Hydrogen Economy (INW-2), Forschungszentrum Jülich, Marie-Curie-Str. 5, 52428 Jülich, Germany; ⊥ Department of Chemistry and Biochemistry, University of California Los Angeles, Los Angeles, California 90095, United States

**Keywords:** impedance spectroscopy, grand-canonical density functional theory, electric double layer, Helmholtz capacitance, polarizability

## Abstract

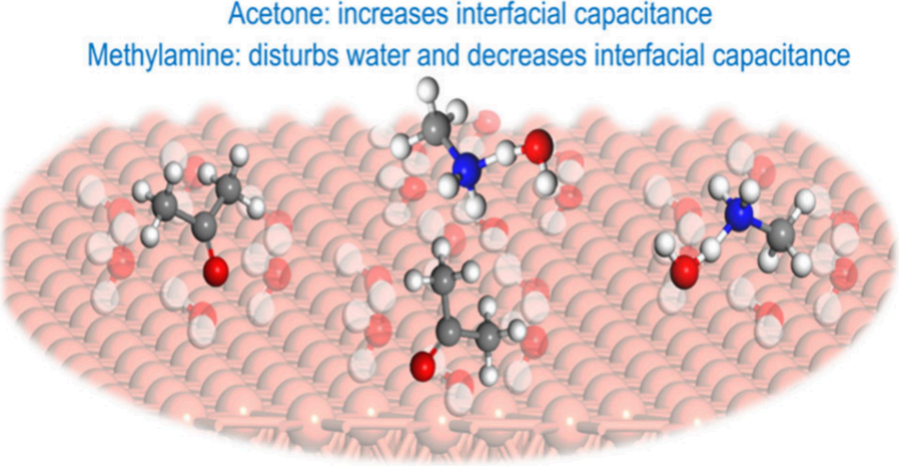

To elucidate interfacial dynamics during electrocatalytic reactions, it is crucial to understand the adsorption behavior of organic molecules on catalytic electrodes within the electric double layer (EDL). However, the EDL structure in aqueous environments remains intricate when it comes to the electrochemical amination of acetone, using methylamine as a nitrogen source. Specifically, the interactions of acetone and methylamine with the copper electrode in water remain unclear, posing challenges in the prediction and optimization of reaction outcomes. In this study, initial investigations employed impedance spectroscopy at the potential of zero charge to explore the surface preconfiguration. Here, the capacitance of the EDL was utilized as a primary descriptor to analyze the adsorption tendencies of both acetone and methylamine. Acetone shows an increase in the EDL capacitance, while methylamine shows a decrease. Experiments are interpreted using combined grand canonical density functional theory and ab initio molecular dynamics to delve into the microscopic configurations, focusing on their capacitance and polarizability. Methylamine and acetone have larger molecular polarizability than water. Acetone shows a partial hydrophobic character due to the methyl groups, forming a distinct adlayer at the interface and increasing the polarizability of the liquid interface component. In contrast, methylamine interacts more strongly with water due to its ability to both donate and accept hydrogen bonds, leading to a more significant disruption of the hydrogen bond network. This disruption of the hydrogen network decreases the local polarizability of the interface and decreases the effective capacitance. Our findings underscore the pivotal role of EDL capacitance and polarizability in determining the local reaction environment, shedding light on the fundamental processes important for electro-catalysis.

## 1 Introduction

Biomass^1,2^ presents a promising renewable carbon source bearing the potential to create closed carbon cycles,^3^ which is beneficial for greenhouse gas reduction. Levulinic acid is a major platform molecule extracted from biomass.^4^ For the conversion of such platform molecules into value-added products, electrochemical reductive amination offers a green way to selectively introduce nitrogen functionalities into organic compounds, where solid–liquid interfaces are of great importance for understanding and improving the reactivity and selectivity.^5−8^ It is thought that the microscopic structures of interface water and adsorbates in the electric double layer (EDL) can significantly alter the electrocatalytic activity of electrode materials.^9−13^ Therefore, it is vital to elucidate the EDL structures and their dielectric properties (i.e., the capacitance) at the atomic level to improve our understanding of electrocatalysis.

However, electrostatic interactions at solid–liquid interfaces are drastically modified by the screening of the solvent on the one hand and the many-body polarization and charge-transfer due to interactions with the (metallic) surface and the water solvent. As an example, water interacts quite strongly with metals, leading to near-chemisorption, which strongly modifies the hydrogen-bonding between the water molecules themselves.^14^ Impedance spectroscopy measurements^15,16^ can provide double layer capacitance values resulting from the adsorption of organic molecules. In general, the adsorption of organic molecules competes with the adsorption of ions. At the potential of zero charge (*E*_pzc_), the EDL is neutral, which makes it possible to isolate and analyze nonelectrostatic interactions (minimal competition with the adsorption of ions), providing clearer insights into the adsorption mechanism of organic molecules.^15−18^ Thus, the change in double-layer capacity at the *E*_pzc_ can be attributed to the adsorption of the organic molecules.^15,16,19^ However, the knowledge of the change in double layer capacitance obtained from the impedance spectroscopy measurements does not provide any conclusions about which effect or which realistic geometrical configuration of the organic molecules on the electrode surface or close to it causes the observed change. Numerous theoretical works utilize molecular dynamics to simulate the interfacial phenomenon within the EDL in terms of cation effects,^20,21^ electrolyte types,^22^ reactant’s hydration diameter,^23^ electrode material,^24^ etc., and exhibit its important role in describing the environment within EDL that is intimately coupled with the surface reaction energetics and is therefore a critical aspect of the overall system performance.^21,25,26^

In this paper, we used EDL capacitance to understand the reaction environment within the EDL before the electro-catalysis of acetone with methylamine as the nitrogen source. Acetone and methylamine were selected as model compounds to represent important reactants during electrochemical amination rather than using longer-chain ketones or primary amines, as they are the first representatives of their homologous series. Impedance spectroscopy measurements were applied to probe the capacitance at *E*_pzc_ of the EDL forming at different concentrations in an aqueous environment. The surface preconfigurations were determined by analyzing isothermal adsorption curves. These curves were obtained through capacitance measurements and then compared to the capacitance values observed at saturation coverage, providing insights into the adsorption behavior and surface interactions. Theoretical simulations were used for molecular scale modeling of the EDL, including configurations, EDL capacitance variations, and polarizability. Starting from the water layers on the Cu(111) surface, the interactions between the water layers and acetone/methylamine were studied using models with different amounts of explicit water molecules complemented by a dielectric continuum. Specifically, models including one or three layers of explicit water molecules were used. Ab initio molecular dynamics (AIMD) based on energies and forces from density functional theory (DFT) is used to fully include the effects of reorientation of water molecules and the steric or electronic effects of adsorbates. Then grand canonical DFT (GCDFT) with a variable number of electrons around the neutral surface was used to determine the EDL capacitance at *E*_pzc_, which is detailed in the “Methods” (section 4.1.1)

## 2 Results and Discussion

### 2.1 Water/Cu Surface

**Figure 1:**
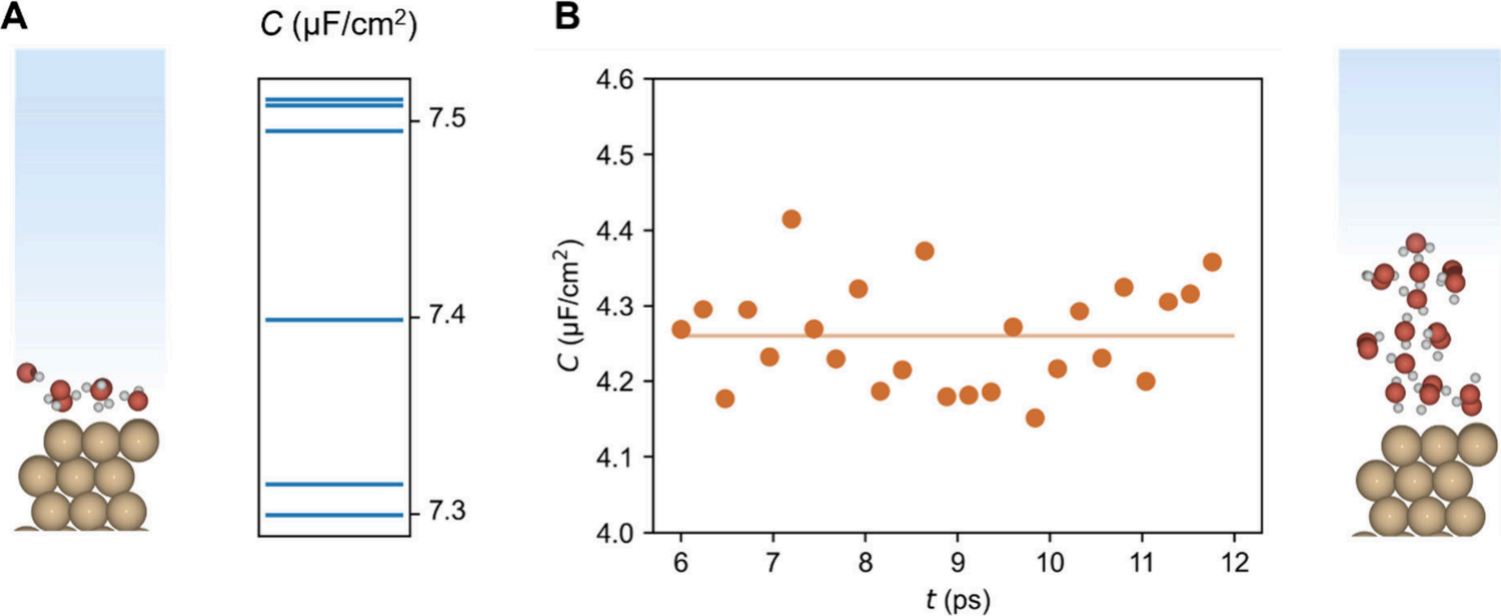
Water/Cu interface is represented by two models: A) Single water layer on Cu(111) with implicit solvation, where the blue lines on the left graph represent the calculated EDL capacitance ranging from 7.3 to 7.5 μF/cm^2^; B) three water layers on Cu(111), also with implicit solvation, where the right graph shows the time evolution of the EDL capacitance (orange dots) from snapshots extracted from an AIMD simulation, with values ranging from 4.2 to 4.6 μF/cm^2^ and the average capacitance indicated by the horizontal orange line.

On the low-index Cu(111) surface, the copper-water interaction is rather weak and the distribution of water molecules in the first hydration shell exhibits a double layer structure with molecules that are closest to the surface mainly located on top of the Cu sites.^7^ Even though a pH of 12 was used, our calculations show that OH^–^ adsorption is unfavorable at *E*_pzc_ (−0.32 V vs RHE), as shown in Figure S1 in the Supporting Information. Therefore, it is safe not to include OH groups at the interface in our study. The atomistic model of the Cu(111)/water interface is shown in Figure 1 including one explicit water layer (left) or three explicit water layers (right), both complemented with a continuum solvent for the bulk water bulk description. For the single explicit water layer model, six configurations were explored, where at *E*_pzc_ the water layer is expected to include equal H down and O down water molecules.^27^ As shown in Figure S2, H-down (defined as situations where a H atom is the lowest atom along the surface normal; see Figure S3) and O-down (defined as situations where a O atom is the lowest, as well as chemisorbed ones) water molecules are equally distributed on the Cu (111) surface. However, they do not exhibit an as ordered “ice-like” arrangement as is commonly observed on Pt surfaces.^7,28^ This is mostly due to the fact that the lattice mismatch between “ice” and Pt is smaller than that in the case of Cu, which has a significantly shorter lattice constant (3.61 Å for Cu and 3.92 Å for Pt). The capacitance was averaged over these six configurations, since they show a very similar free energy (with a difference lower that 0.01 eV) and calculated at around 7.4 μF/cm^2^. The model including three water layers was constructed layer by layer to achieve equilibrium between each layer and underwent an equilibrium process of 12 ps, and then another 12 ps AIMD simulation was applied to consider the dynamic property of water molecules, like reorientation. A snapshot was collected every 240 fs (200 steps) during the second half of the MD trajectory. To sample the EDL capacitance around *E*_pzc_, for each snapshot we have applied the surface charging method for the given geometry, including an implicit solvent (with a dielectric constant of ε = 78.4) and electrolyte in addition to the three explicit water layers. The average capacitance is around 4.2 μF/cm^2^. As shown in Figure S4, as the number of layers of explicit water increases from one to four, there is a decrease in the calculated EDL capacitance, from 7.4 to 4.2 μF/cm^2^, while the value for implicit solvation only, with the dielectric constant of 78.4, is 13.35 μF/cm^2^ (Figure S5). The electronic component for the dielectric constant of water is rather small (∼2) as illustrated by the low dielectric constant of ice, implying that nuclear motions, such as water reorientation, are key for the dielectric response of water. This explains the decrease in the calculated capacitance when more explicit water layers are introduced, since in the snapshots those explicit molecules’ geometries are frozen in the configurations obtained at *E*_pzc_. Besides, the AIMD trajectory shows the motion of interfacial water layers is markedly hindered (see Figure S6). Additionally, H-down and O-down water molecules are, on average, equally distributed within the water film.

The decrease in the dielectric constant of water at interfaces has been previously described. Experiments from Fumagalli et al. showed the presence of an interfacial layer with a very small (∼2) dielectric constant,^26^ with a thickness of 7.4 Å which is very close to that of three molecular layers. This justifies the hybrid model that we use for the calculation of the capacitance with three explicit layers of water to describe the interface region, complemented by an implicit dielectric model to represent the rest of water. The frozen geometry for each MD snapshot models the hindered water mobility at the interface, while the continuum model provides the response of the free water solvent further away from the interface. In the implicit solvation model, the solvent is treated with a high dielectric constant continuum medium (ε ≈ 78.4 for water), which leads to a higher effective capacitance because the system can be polarized more readily in response to changes in surface potential. This high relative dielectric constant is mostly due to the reorientation of water dipoles in the explicit water bulk. However, when static explicit water molecules are introduced at the interface, their electronic dielectric response is much lower compared to the orientational response of bulk water. This results in a reduced ability to screen the electric field at the interface, which leads to a lower effective capacitance.

The model that Stern proposed for the EDL at an aqueous interface emphasized that the dielectric constant is reduced over a nanoscopic width.^29^ Moreover, such a dielectric constant reduction for a dipolar fluid has been related to molecular ordering and orientation using approximate statistical mechanical methods.^30^ Besides, Bonthuis et al.^31^ studied both parallel and perpendicular interfacial dielectric response functions of aqueous interface and demonstrated that the perpendicular dielectric function exhibits singularities like the nonlocal bulk dielectric function,^32^ indicating anomalous screening effects at the interface. Within the water films on the electrode, molecular reorientation is minimal and the electronic response dominates, which is comparable to the high-frequency regime. Similarly, polarizability becomes particularly relevant when discussing high-frequency measurements in electrochemical impedance spectroscopy (EIS). At high frequencies, molecular reorientation is limited, making polarizability responsible for the observed capacitance changes. As an example, the high-frequency dielectric constant of water is about ∼2, as opposed to the low-frequency dielectric constant of ∼78.4. This difference has already been pointed out to be crucial for understanding the capacitance of aqueous interfaces.^21,26,33−37^ Therefore, in the following discussion on EDL capacitance involving adsorbates, polarizability is reported to achieve a microscopic understanding of the observed trends in capacitance.

By comparison with experiments,^26^ the model of three water layers was chosen for calculations including solvated adsorbates. The one monolayer model was also kept for comparison since it allows the counter charge of the model continuum electrolyte to come closer to the surface and therefore better describes the double layer.

### 2.2 Acetone

**Figure 2:**
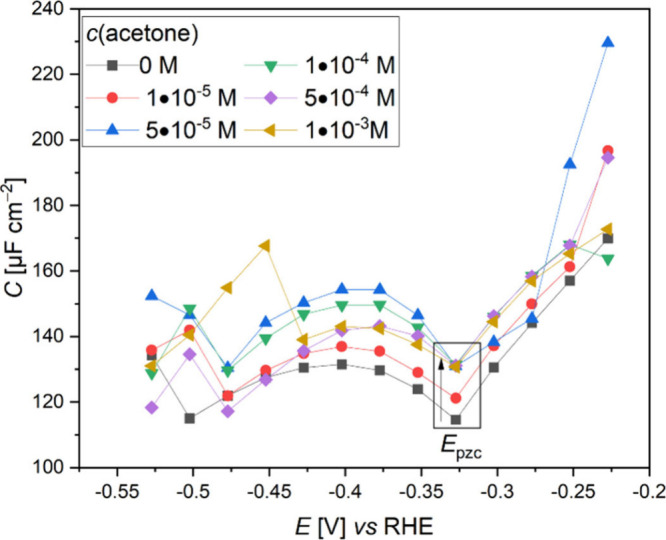
Capacitance vs potential plots on Cu when different acetone concentrations are added to 0.01 M NaClO_4_ at a pH of 12.

The consequence following the addition of acetone to the electrolyte was determined using impedance spectroscopy measurements via electric double layer capacitance (Figure 2). It was found that at *E*_pzc_, there is an increase in the EDL capacitance as the concentration of acetone increases up to a threshold, which was identified as two molecules per 57.9 Å^2^ electrode surface area in our computational study.

**Figure 3:**
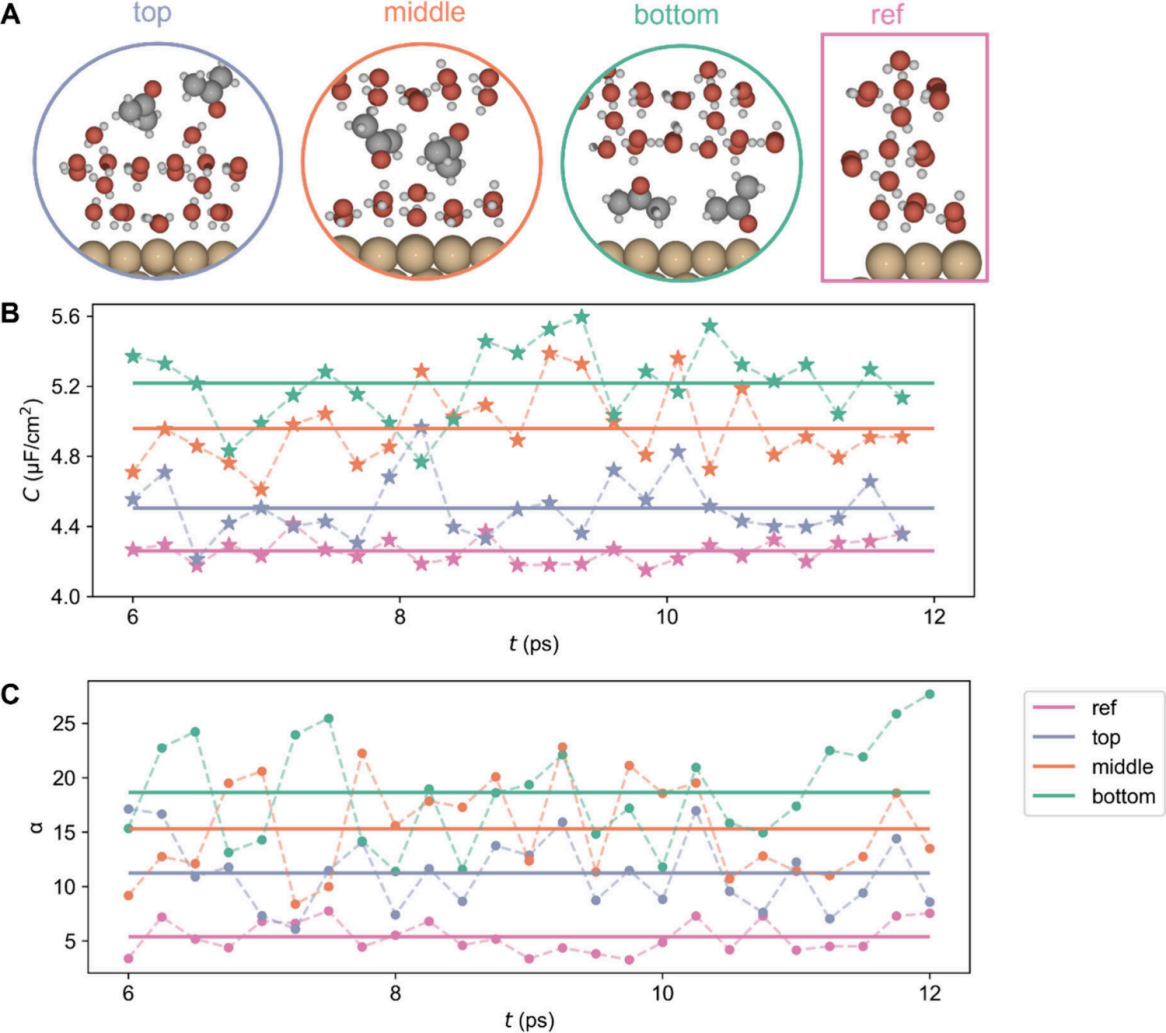
Hybrid solvation models for acetone on Cu(111), utilizing a model with three explicit water layers as a reference. All models are completed by a continuum dielectric to model the water bulk. A) Structure, where a layer of acetone molecules replaces one of the water layers: top (purple), middle (orange), or bottom (green). These models are correspondingly named top, middle, and bottom. B) Calculated electric double layer capacitance during the second half of the AIMD simulations, with sampling points taken every 200 steps (i.e. every 240 fs); C) calculated polarizability along the trajectories, every 200 steps (i.e., every 240 fs) during the second half of the simulations for the three structural models of A and for the reference system of three water layers.

Theoretical microscopic models were constructed to understand the environmental changes after the introduction of acetone. Acetone is a polar molecule with a dipole moment of 2.88 D, soluble in water, but that also presents some hydrophobic nature due to its methyl groups.^38,39^ Moreover, it has been reported in many experimental^40−42^ and theoretical works^43,44^ that the dielectric medium inside the Helmholtz layer may not be as homogeneous as that represented in the traditional model. Based on these insights, acetone models start with the three-layer explicit water structure (Figure 3A) and replace one water layer with a layer of acetone molecules, as shown in structures in Figure 3A. These models have the purpose of representing the local environment surrounding of the acetone molecules, for different positions with respect to the surface: far (top layer), intermediate (middle layer), or in direct interaction with the surface (bottom layer). The adsorption behaviors of acetone on Cu(111) were detailed in Figure S7.

AIMD was applied first to consider the water reorientations with the addition of acetone molecules and the corresponding translation and rotations for acetone molecules. After an equilibrium process of 12 ps, another AIMD at room temperature was used to simulate the dynamic properties and sample the EDL capacitance (Figure 3B) and polarizability (Figure 3C). Along with the trajectories, the molecular arrangement in the models is not changed, with acetone molecules remaining in the same layer with no molecular exchanges between layers (Figure S8). All configurations sampled along the AIMD trajectory result in an increase of the EDL capacitance, which is consistent with the measurements from experiments. To be specific, when acetone is in the bottom layer, the capacitance increases the most, followed by the model with acetone in the middle layer. The polarizability results show the same trend as capacitance. It indicates that the introduction of acetone molecules will also increase the polarizability at the interface. The molecular polarizability of acetone is reported as 7.9 ± 0.2 Å^3^,^45^ while that for water is around 1 Å^3^.^46^ Therefore, when acetone is introduced to water layers, this molecule not only repels water molecules in the local environment but also contributes to the higher polarizability within the interface. The potential at zero charge *E*_pzc_ for the snapshots selected along the MD trajectory is shown in Figure S9. Acetone, as an electron-withdrawing group, promotes charge transfer from the Cu electrode surface to the adsorbates, leading to a slightly more positive *E*_pzc_. Given the rather small lateral size of our unit cell, this shift in *E*_pzc_, which is not seen in experiment, is likely attributable to finite size effects, where larger unit cells would be required to sample diverse lateral acetone arrangements.

As shown in Figure S10, one can note that after the introduction of acetone into the water layers there is still an equal distribution between H-down and O-down water molecules, which indicates that acetone does not interact strongly with the water hydrogen bonding network. A higher polarizability in the medium increases the dielectric constant and enhances the amount of charge stored at the interface for a given applied potential, therefore increasing the capacitance.

**Figure 4:**
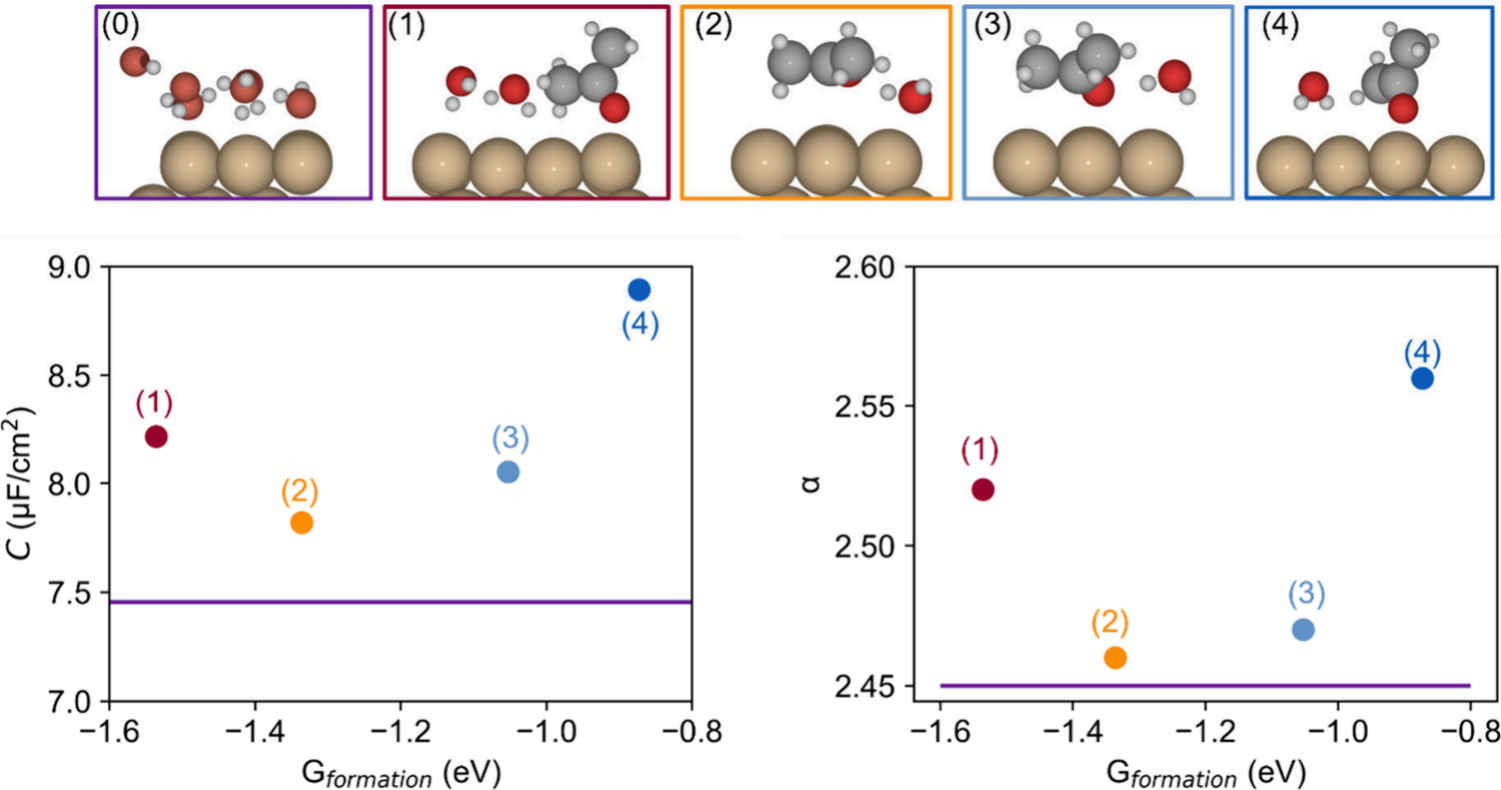
One-layer models for acetone adsorption at the Cu(111)-water interface. Calculated capacitance (left) and polarizability (right) as functions of the formation energy. The colors of the points correspond to that of the frame for the structures. Red: one vertically adsorbed acetone molecule and two water molecules oriented hydrogen-down; orange: one parallel adsorbed acetone molecule and a water molecule oriented oxygen-down; light blue: same as orange but with the water molecule oriented hydrogen-down; dark blue: vertically adsorbed acetone molecule with a hydrogen-down oriented water molecule; purple: single water layer as reference.

We now move for comparison to the model with one explicit water layer, complemented with an implicit solvent, where we have sampled several structures for water and acetone on the surface (Figure 4). There are two adsorption modes of acetone on the 3 × 3 Cu (111) model, the parallel one and the vertical one, as shown in Figure S7. A single acetone molecule was considered in our 3 × 3 unit cell to study the interactions between acetone, water molecules and the Cu (111) surface. When acetone adsorbs vertically on the Cu (111) surface via the O–Cu bond, the other water molecules cannot adsorb through the O-down model, and at maximum two water molecules have space to accompany acetone, as shown in the red (structure 1) and dark blue labeled structures (structure 4). At the same time, if acetone adsorbs in a parallel manner, the remaining space is limited to one water molecule, but this water molecule can be in the mode of H down (light blue) and O down (orange). All these structures yield an increase of the capacitance compared with the reference system, including one explicit water layer. The calculated polarizability provides a very similar trend, confirming that polarizability is the main descriptor for the EDL capacitance and also the local environment.

It can be noticed, by comparing Figures 3 and 4, that the value of the polarizability and the amount of change of that polarizability for a given change in the EDL capacitance are both much larger for the three-explicit-water layer model compared to the one-layer model. For example, as shown in Figure 3B, taking acetone in the bottom layer model as an example, the EDL capacitance increases by 21%, while the polarizability increases 200%. However, when it comes to the one-layer model, taking structure 1 as an example, capacitance increases by 11% while polarizability increases by 16%. In brief, in three-layer models, the polarizability increase degree is very significant. The substantial change in polarizability between configurations for the three-layer model can be explained by a more pronounced collective effect. First, the density of acetone is high in the model with two acetone molecules per layer. Second, the other layers of explicit water molecules also respond electronically, hence enhancing the effect. It is important to note that despite the high density of acetone, the hydrogen bond network of water is not significantly disrupted, allowing the water molecules to maintain their collective electronic response.

### 2.3 Methylamine

**Figure 5:**
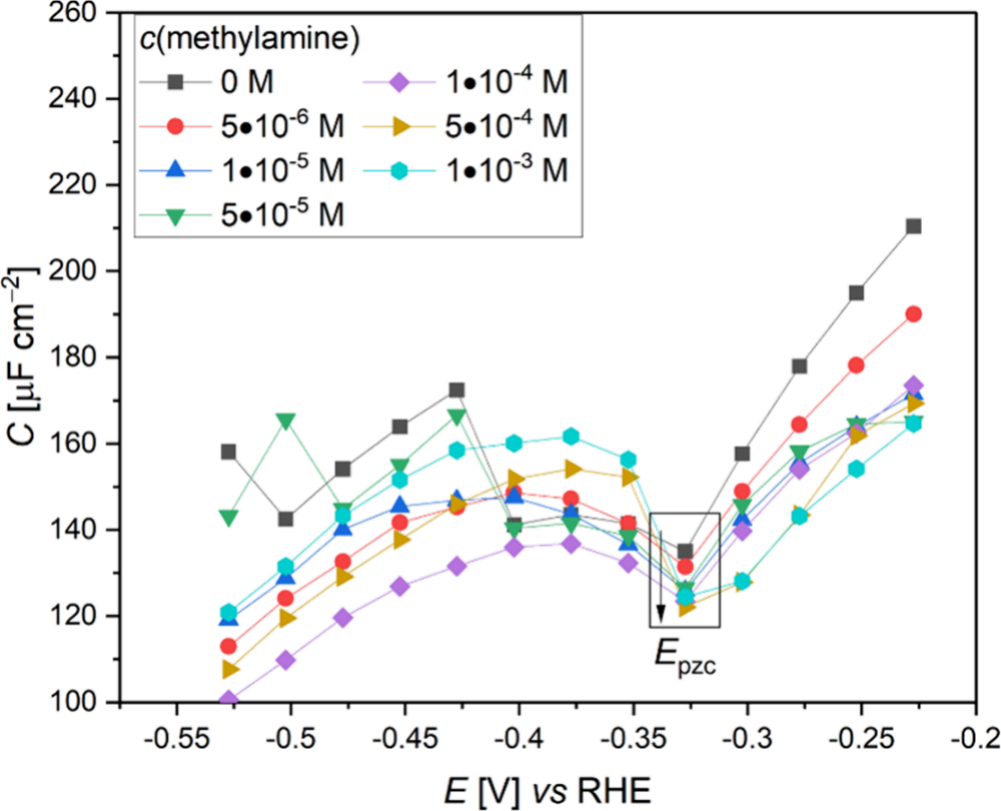
Capacitance vs potential plots on Cu when different concentrations of methylamine are added to 0.01 M NaClO_4_ at a pH of 12.

In contrast to acetone, at *E*_pzc_, the introduction of methylamine at the water/Cu interface results in a decrease of the capacitance (Figure 5), where the higher the concentration of methylamine, the lower the capacitance.

**Figure 6:**
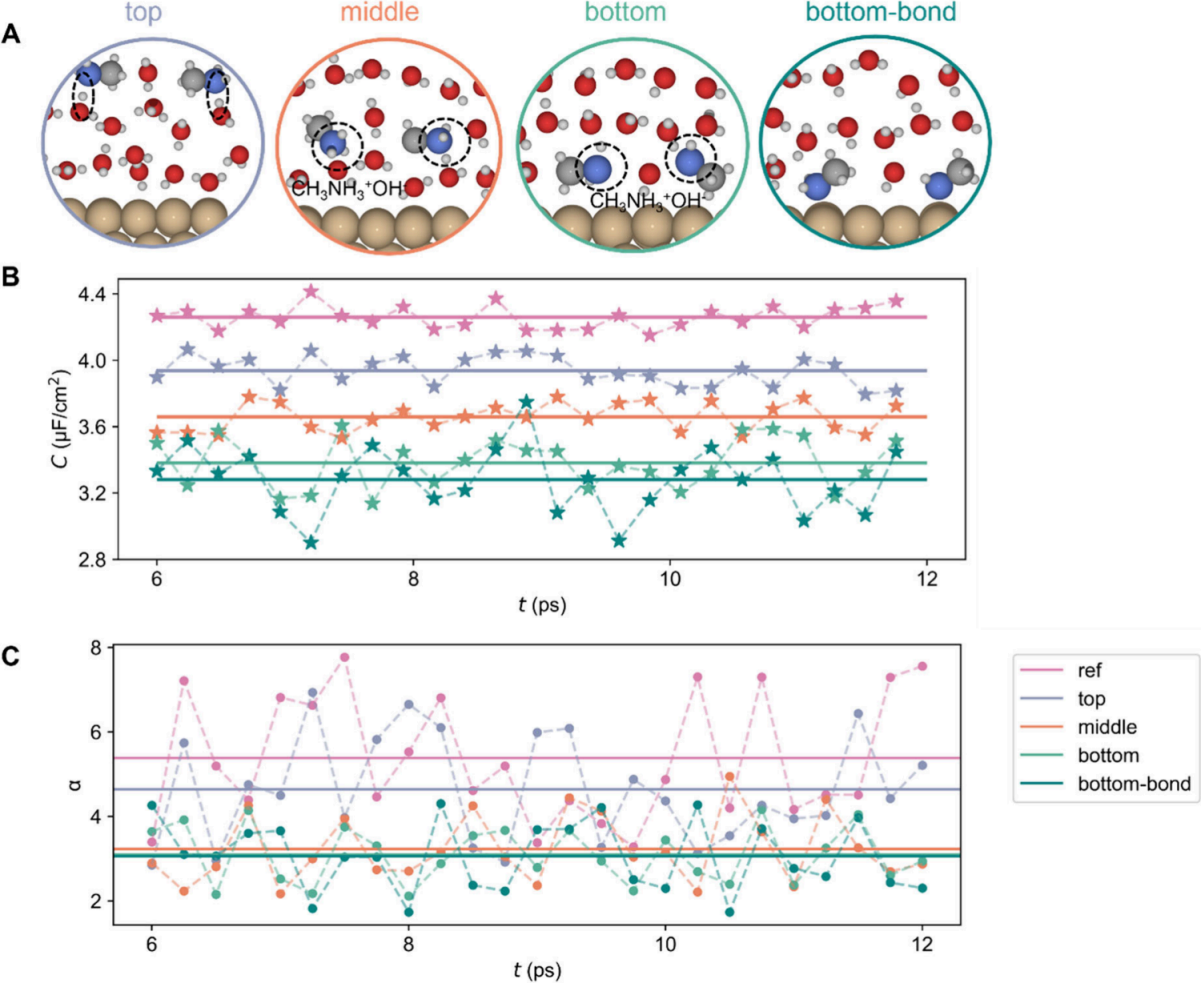
Hybrid solvation models for methylamine on Cu(111), utilizing a model with three explicit water layers, as a reference. All models are completed by a continuum dielectric to model the water bulk. A) structure, where two methylamine molecules are combined with two water molecules in the top (purple), middle (orange), or bottom (green), and these models are correspondingly named top, middle, and bottom. B) calculated electric double layer capacitance during the second half of the AIMD simulations, with sampling points taken every 200 steps (i.e., every 240 fs); C) calculated polarizability along the trajectories, every 200 steps (i.e., every 240 fs) during the second half of the simulations for the three structural models of A and for the reference system of three water layers.

Methylamine is also polar with a dipole moment of 1.31 D.^11^ Besides, the amino group (−NH_2_) of methylamine (CH_3_NH_2_), can promote the hydrolysis of water, forming CH_3_NH_3_^+^OH^–^ adducts. Methylamine is somewhat larger than water and this triggers the question of the number of water and methylamine molecules in one layer on the (3 × 3) unit cell. Based on our adsorption studies of methylamine on the 3 × 3 Cu(111) surface (Figure S13), we considered the combinations of one methylamine with four water molecules, two methylamines with two water molecules, and three methylamines with one water molecule. The models for two methylamines with two water molecules are shown in the structures in Figure 6, placing the methylamine molecules in either the bottom, middle, or top layers from our three-explicit-water-layer model. When it comes to the methylamines in the bottom layer, the competition between methylamine and water in terms of adsorbing on the surface was considered, constructing two models with bonded methylamine via N–Cu bond and nonbonded methylamine.

AIMD was applied to fully consider the reorientations of water molecules and the translation and rotation of methylamines. Corresponding EDL capacitance and polarizability were analyzed based on the second half of the 12 ps AIMD simulation at room temperature after another equilibrium process of 12 ps. The spontaneous formation of CH_3_NH_3_^+^OH^–^ can be seen specifically in the models with methylamine in the middle or bottom layers, whereas when methylamine molecules are placed in the top layer, there is one water molecule pointing toward methylamine via one hydrogen. Along the trajectories, the molecular arrangement in the models is not changed, with methylamine molecules remaining in the same layer with no molecular exchanges between layers (Figure S14). All structures show a decrease in capacitance compared with the reference system, with three explicit water layers. Models with the methylamine in the bottom layer provide the largest decrease in the calculated capacitance, and they also correspond to better stability (Figure S15, where the last snapshot of AIMD was used to do geometry optimization). The polarizability results show the same trend as well, with a decreased value in the presence of methylamine, even though methylamine has a higher individual molecular polarizability than water. This is explained by the fact that methylamine disrupts the hydrogen bonding network of water more than it contributes through its own polarizability. As shown in Figure S17, the introduction of methylamine into the water layers disturbs the distribution of H-down and O-down water molecules, leading to a dominance of H-down orientations. This structural arrangement is evident in Figure 6A, where the water molecules surrounding methylamine, whether positioned above, below, or within the same layer, prefer the H-down adsorption modes. This is because methylamine molecules tend to be protonated from near water molecules, forming CH_3_NH_3_^+^OH^–^ ion pairs at the interface. Therefore, methylamine, whether in its neutral or protonated form, induces a hydrogen bonding network with the surrounding water molecules, which leads to a specific ordered arrangement with more H-down water molecules around methylamine molecules compared to the pure Cu/water interface (Table S2 and Figure S17). The H-down orientation of the water molecule is expected to lead to a weaker coupling with the surface compared with O-down water molecules which interact with the surface via a stronger O–Cu bond. Moreover, as shown in Figure S2, H-down water molecules result in a less polarizable interface (α = 2.05) compared with O-down water molecules (α = 2.44). Therefore, this shift toward more H-down orientations disrupts the initial water network, decreases the overall water polarizability at the interface, and ultimately reduces the capacitance. Even though from the above discussion we observed a positive correlation between EDL capacitance and polarizability, the ultimate molecular underpinning is not clear yet: The exact connection between the molecular orientations, the intermolecular interactions, and the resulting polarizability (and capacity) is beyond the scope of this study.

The potential at zero charge was also determined for the snapshots selected along the MD trajectory from the GCDFT calculations (Figure S18). Unlike acetone, the introduction of methylamine results in a slightly more negative potential. This behavior can be attributed to the fact that methylamine acts as an electron-donating group, leading to an increased electron density on the surface.

**Figure 7:**
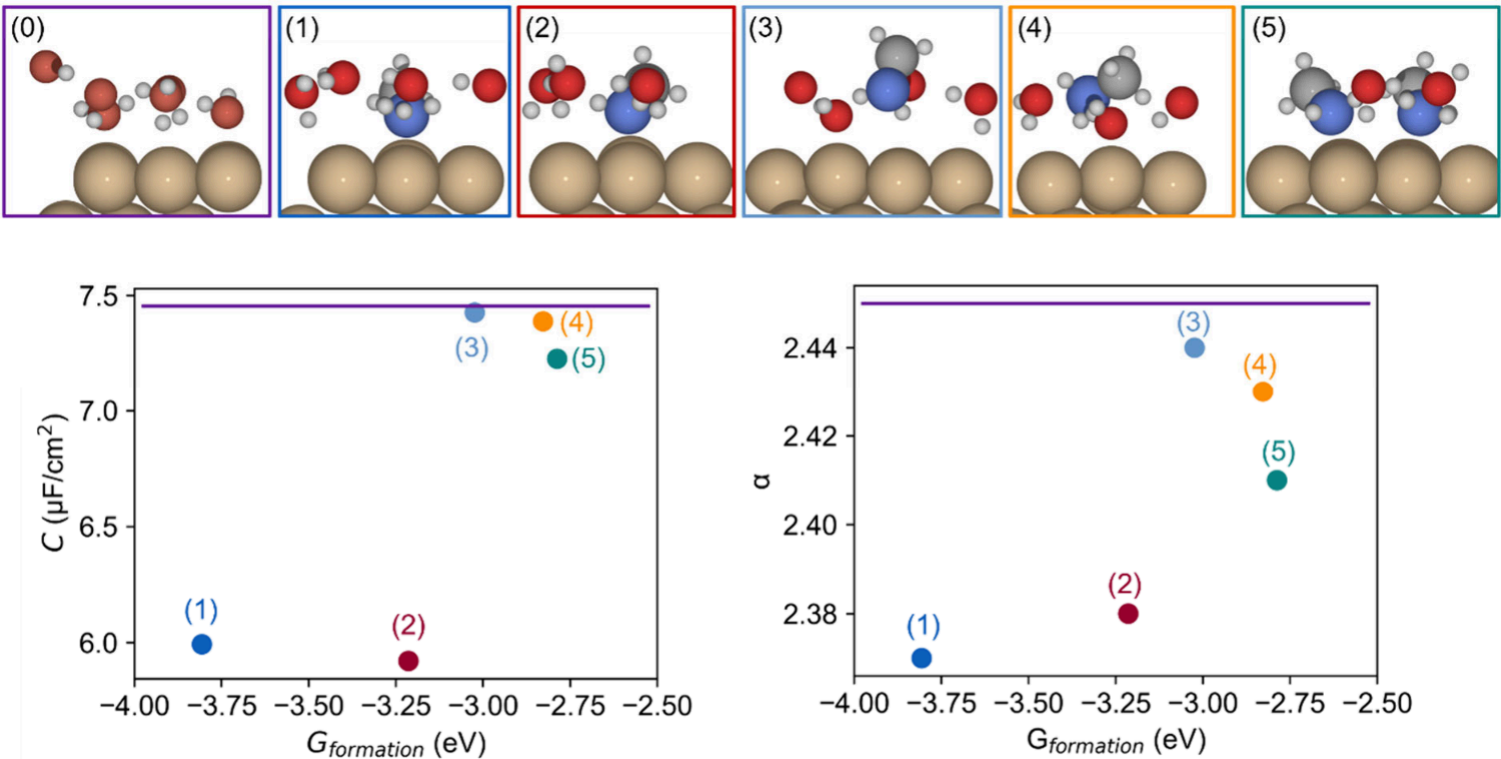
Diagram illustrating a sequence of structures for a monolayer mixture of methylamine and water molecules, ranked from the most stable to the least stable configuration: purple is used for the reference system, which consists of a single water layer; dark blue represents a structure with one nitrogen-bonded methylamine molecule surrounded by four confined water molecules; red shows a similar configuration but with three water molecules; light blue depicts a nonbonded methylamine with four water molecules; orange illustrates a structure with a nonbonded methylamine and three water molecules; green denotes a configuration with two methylamine molecules paired with two water molecules. Accompanying the structural representations are graphs displaying the corresponding capacitance (left) and polarizability (right) versus their Gibbs formation energies, effectively correlating structural stability with physical properties.

For comparison, monolayer models similar to those for acetone are constructed (Figure 7). Considering the adsorption behavior of methylamine on a 3 × 3 Cu(111) surface, monolayer structures presenting one or two methylamines with water molecules are investigated. All considered configurations show a decrease in capacitance compared to the reference system, one water layer. The calculated polarizability is also decreased and shows a very similar trend compared to the capacitance. The same phenomenon occurs as in the previous three-layer models, the introduction of methylamine, despite its inherently higher polarizability compared to that of water, significantly disrupting the hydrogen bonding arrangement of water molecules at the interface. This disruption leads to reduced flexibility and responsiveness of the system’s electron cloud. The decrease in polarizability is, however, much smaller in the case of the one-layer model due to the reduced number of water molecules that are affected by the presence of methylamine.

It is interesting to note that the carbonyl, known as an electron-withdrawing group, is oriented toward the Cu surface at the maximum increase of capacitance (as seen in panel 4 of Figure 4), while the amine, known as an electron-donating group, is oriented toward the Cu surface at the maximum decrease of capacitance (as shown in the bottom-bond configuration in Figure 6). These observations suggest a link between molecular orientation, charge-transfer and the property of the Cu/solvent interface. The corresponding Bader charge analysis is provided in Figure S19 and Figure S20. However, determining a clear trend or causal relationship between molecular orientations and capacitance would require a more comprehensive statistical analysis and further studies. Future analysis involving projected density of states (DOS) might provide deeper insights into the charge (re)distribution and interactions, helping to elucidate the origin of these effects.

For ideal capacitors, polarizability and capacitance are related, as both are second-order derivatives of energy: capacitance with respect to charge and polarizability with respect to the electric field. This connection is reflected in the parity plots (polarizability as a function of capacitance) provided for both acetone (Figure S21) and methylamine models (Figure S22), where we see that polarizability and capacitance show similar global trends. However, despite their overall alignment, the evolution of capacitance and polarizability is not linearly correlated. These differences can be attributed to several factors. First, we must keep in mind that polarizability is analyzed without considering the implicit solvent. This contrasts with the capacitance, where the implicit solvent contributes substantially. In general, the polarizability seems to vary more significantly than the capacitance within a series of related systems. This is likely due to the vibrational degrees of freedom of the interface sampled at 300 K: Stretched and compressed bonds change the polarizability of the system that is not large enough to average them out within a single snapshot. However, the capacitance is less sensitive to such minute changes as the stabilization of the surface charge dominates.

## 3 Conclusions

Our study systematically explored the interactions between acetone, methylamine, and the copper electrode within the electric double layer (EDL) in an aqueous environment, providing significant insights into the electrochemical amination process. Key findings include the distinct roles of acetone and methylamine in modulating the structure and properties of the EDL. Capacitance was used as a primary descriptor to quantification of these interactions and the subsequent impact on the electrode’s surface chemistry. The two molecules provide a different trend for the change in EDL capacitance, with an increase for acetone and a decrease for methylamine. Our integration of ab initio molecular dynamics (AIMD) for the structure and grand canonical density functional theory (GCDFT) for the capacitance provided a detailed microscopic view of these processes, offering predictions on the polarizability and capacitance changes induced by the presence of reactants. When acetone is introduced at the EDL interface, calculations show an increase in both capacitance and polarizability. An acetone molecule is more polarizable than a water molecule. At the interface, acetone, which presents some hydrophobic character from its two methyl groups, repels water and contributes to a higher polarizability and hence a higher capacitance. Models with three or one explicit layer of water molecules provide qualitatively similar results, although models including three layers provide a markedly larger polarizability increase. Methylamine interacts more strongly with the hydrogen bonding network of water and can be protonated when it does not bind directly to the Cu surface. Although methylamine also has a higher individual molecular polarizability than water, its main effect is to disrupt the hydrogen bonding network of water and to decrease its ability to be polarized at the interface. These results not only advance our understanding of the fundamental aspects of electro-catalysis but also highlight the crucial influence of EDL characteristics in optimizing the conditions for electrochemical reactions. By elucidating the role of molecular adsorption and interfacial properties, this research paves the way for more effective control and enhancement of the catalytic performance in electrochemical systems. Future studies could further refine this approach by examining the role of solvated ions in electrolytes in the EDL structures, thereby confirming the applicability of these findings to a broader range of electrochemical processes.

## 4 Methods

### 4.1 Computational Details

The Vienna Ab initio Simulation Package (VASP) was employed for all periodic DFT calculations.^47^ The Perdew–Burke–Ernzerhof (PBE) functional within the generalized gradient approximation (GGA) was used for the exchange-correlation, along with the dDsC dispersion correction to account for van der Waals interactions.^48,49^ The energy cutoff was set to 400 eV. Interactions between atomic cores and electrons were modeled using the projector augmented wave (PAW) method.^50^ Initial geometry optimizations were performed in vacuum until the forces on each atom were below 0.01 eV/Å and the total energy converged to less than 10^–6^ eV. Subsequently, geometry optimizations using implicit solvation via VASPsol^51^ were conducted, with convergence criteria of 0.02 eV/Å for forces and 10^–6^ eV for energy.

A three-layer (3 × 3) supercell of Cu(111) termination was used, where the bottom layer was fixed. A vacuum slab of 15 Å thickness was added in the *Z* direction. When it comes to solvation optimization and Surface Charging, all structures are symmetrized, and the box thickness in the *Z* direction is 60 Å for the implicit solvation region. To be specific, five layers were used with water layers and/or adsorbates on both sides. The Brillouin zone was sampled using 5 × 5 × 1 Gamma-centered *k*-point grids for geometry optimization. The model with three water layers is constructed layer by layer to achieve equilibrium within and between layers.

#### 4.1.1 GCDFT Simulations of Solvated Models on Cu(111)

Grand canonical density functional theory (GCDFT) was used to evaluate the free energy of the solvated models on Cu(111). Details can be found in our previous work,^52−55^ and here we summarize the key points with respect to the Helmholtz capacitance.
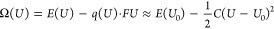
1

Here, *E*(*U*) is the electronic energy of the surface under potential *U*, which is calculated by referencing the Fermi level of the system against the vacuum level. *q*(*U*) is the surface charge difference referenced against the neutral system, and *F* is the Faraday constant. *U*_0_ stands for the potential of zero charge in the vacuum scale, and *C* is the effective capacitance. This method, which treats the electrochemical interface as an effective capacitor, allows us to calculate the Helmholtz capacitance based on the electronic energy and surface charge differences at varying potentials, as described by eq 1. The polarizable electrolyte region is modeled using the linearized Poisson–Boltzmann implicit solvation model in VASPsol, with parameters such as the dielectric constant of water (78.4) and Debye screening length (0.1 M concentration) set to represent realistic conditions.

Snapshots were extracted from the MD trajectory of the three-water layer model, and the corresponding capacitance for each geometry was derived from the curvature of the energy-potential parabola obtained by using the surface charging technique. In this case, the structure was frozen, which captures the short-time electronic response but not the long-time structural reorientation effects. This corresponds to our hypothesis that the motion of the first three water layers is hindered at the interface with the metal, so that only their electronic degrees of freedom contribute to the capacitance, while other layers are represented by the continuum solvent with bulk dielectric constant. This procedure is expected to be relevant for the capacitance around *E*_pzc_, as our AIMD simulations are performed for neutral surfaces and, thus, capture the thermal fluctuations of the molecular orientations under these conditions. Further away from *E*_pzc_ we would expect more significant contributions of molecular reorientations to the low-frequency, long-time scale capacitance.

The surface free energy was calculated based on the grand canonical free energy to study the stability at *E*_pzc_.
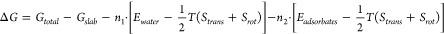


The translational and rotational entropy of liquid water and adsorbates in solvation is approximated as half the value for the ideal gas.^56^

#### 4.1.2 Electric Field Calculations

Electric fields were applied using EFIELD parameters in VASP and using asymmetric systems. This parameter controls the magnitude of the applied linear electric field. Dipole corrections to the potential (LDIPOL = .TRUE.) are turned on to avoid interactions between the periodically repeated images. The electric field was applied in the *Z* direction (IDIPOL = 3). A set of potentials ranging from −0.5 to 0.5 eV/Å were used. At each magnitude of the applied electric force field, the selected snapshot geometries were kept frozen to obtain their polarizability. The energy variation as a function of the applied electric field is a quadratic function:
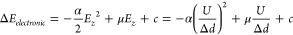
where Δ*E*_*electronic*_ means the electronic energy difference at different electric fields compared to that under zero electric field; *E*_*z*_ is the electric field magnitude in *z* direction; α is the polarizability; μ is the dipole moment; *U* is the applied potential; Δ*d* is the thickness of the electric double layer; *c* is a constant.

Electrostatic potential data across the system is provided in Figure S11 for acetone and Figure S16 for methylamine to demonstrate that the interactions are cut off at the boundaries of repeated images of periodic cells.

#### 4.1.3 AIMD Sampling of Thermal Fluctuations within Solvated Models

AIMD simulations provide insight into the dynamic response of the system, including the dipole reorientation of molecules in the solvated region. Ab initio molecular dynamics simulations were performed by using the VASP program. Optimized structures with the same DFT settings were selected as the initial configurations. AIMD simulations were initially run in the *NVT* canonical ensemble at a temperature of 400 K, and the time step was set to 1.2 fs. Trajectories of 12 ps (10000 steps) were collected. After that, the *NVT* canonical ensemble at room temperature was used to obtain another 10 ps trajectory. From those simulation of 10000 steps, a point is collected every 240 fs (every 200 steps) during the second 5000 steps to calculate their averaged Helmholtz capacitance using GCDFT.

### 4.2 Experimental Details

#### 4.2.1 Impedance Spectroscopy

##### 4.2.1.1 Chemicals

Methylamine (40%) and sodium perchlorate monohydrate (≥99%) were purchased from Merck. Acetone (>99.5%) was purchased from Th. Geyer, while abcr was the supplier of the utilized potassium hydroxide (85%).

##### 4.2.1.2 General Procedure

Before each electrochemical measurement, the copper electrode was wet sanded with 1000 and 2000 grit to remove organic impurities or metal oxides from the surface. The electrochemical active surface of the electrodes was limited to 1 cm^–2^. The copper electrode was used as a working electrode (WE). As a counter electrode (CE), platinum was utilized. All electrochemical measurements were performed in a 20 mL vial. The WE, CE, and reference electrode (Hg/HgO in 1 M KOH) were fixed to a 3D printed cap that sealed the vial. The electrolyte, a 0.01 M NaClO_4_ solution, was prepared with Milli-Q water and adjusted to a pH value of 12 by adding KOH. Then acetone or methylamine was added to the electrolyte solution to obtain the desired concentration. After homogenization of the mixtures, electrochemical measurements were performed at room temperature.

The electrochemical measurements were performed using a *Metrohm* Autolab B.V. PGSTAT 302N equipped with the impedance module FRA32M. The impedance was measured at frequencies from 0.1 to 1000 Hz (in total 40 frequencies) in 25 mV steps from −0.227 to −0.527 V vs RHE. The potential was applied two min prior each impedance measurement to generate an equilibrium at the electrode surface.

**Figure 8:**
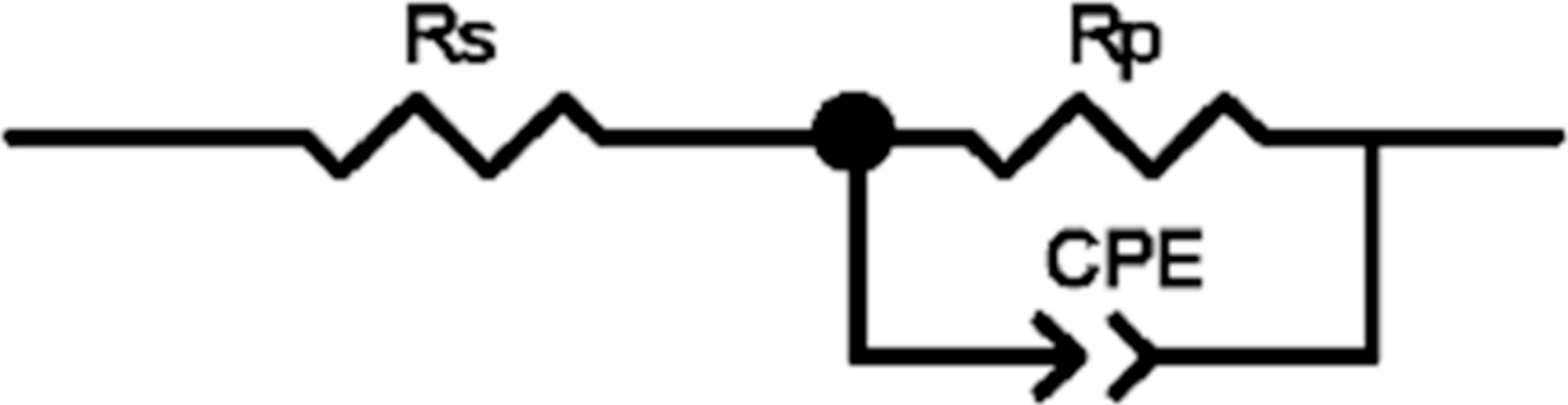
Utilized equivalent circuit to fit the impedance data.

**Figure 9:**
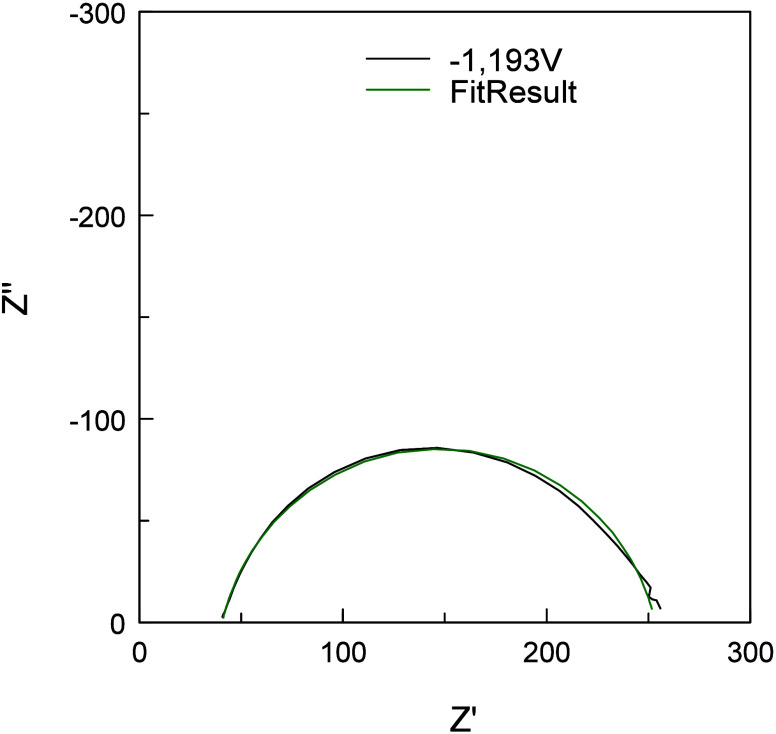
Example of the measured impedance data and the resulting fit after applying the equivalent circuit to the measured data. Conditions: 0.01 M NaClO_4_, pH = 12, measured at −1.193 V vs Hg/HgO ≙ −0.327 V vs RHE ≙ *E*_pzc_, 0.1–1000 Hz (40 frequencies in total).

The copper electrode did not behave like a pure capacitor, as semicircles were measured.^57^ For determining the double layer capacitance, the data was first fitted to a modified version of the simplest RC equivalent circuit, replacing the double layer capacitor with a constant phase element (CPE) (Figure 8).^57^ The fitting of the data was done by using *ZView* for *Windows*. The CPE considered, for example, a certain surface roughness and its heterogeneity which might impact the impedance data.^57^ As shown in Figure 9, the fit described the measured data well, illustrating that the equivalent circuit used is a good descriptor of the system. Finally, the double-layer capacity was calculated by applying eq 2. The charge transfer resistance *R*_p_ between the electrode and the solvent required for eq 2 was obtained from the fit of the experimental data with *ZView*, while the frequency used is the one that describes the vertex of the semicircle best. The final *C* value was obtained after normalizing *C*_dl_ with respect to the geometric active surface (eq 3).
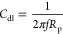
2where *f* is frequency [Hz], *R*_p_ is charge transfer resistance [Ω], *C*_dl_ is the double-layer capacity.
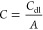
3where *A* is the electrochemical active surface (geometric) [cm^2^].

## Data Availability

The data that support the findings of this study are available from the corresponding author upon reasonable request.

## Supporting Information Available

Calculated OH^–^ adsorption Gibbs free energies, distribution of H-down and O-down water molecules within one water layer and acetone three-layer model, classification of water molecules’ orientations, capacitance and corresponding Gibbs formation energies, bare Cu(111) under implicit solvation model, mass center of water molecules and the distribution of H-down and O-down water molecules, adsorption modes of acetone and methylamine on 3 × 3 Cu(111) surfaces, mass center changes of acetone, methylamine, and water layers, variation of *E*_pzc_ for three-layer acetone and methylamine models, local potential along *z* direction for acetone and methylamine models, formation energy for each acetone and methylamine three-layer models, average value and standard deviation along the trajectory for the potential of zero charge *E*_pzc_ during the AIMD simulation, parity plots, average and RMSD (root mean square deviation) of water orientations during AIMD simulation (PDF)
