# Supplementary material for: Probing the Electric Double-Layer Capacitance to Understand the Reaction Environment in Conditions of Electrochemical Amination of Acetone
Source: ACS Appl Mater Interfaces. 2025 Jan 2;17(2):4087–97. doi: 10.1021/acsami.4c14134 (PMC11744509; doi:10.1021/acsami.4c14134)
Supplement: Supplementary file 1 — am4c14134_si_001.pdf [file am4c14134_si_001.pdf]

Supporting Information

Probing the Electric Double Layer Capacitance to Understand the Reaction Environment in Conditions of Electrochemical Amination of Acetone

Yani Guan,[a], # Justus Kümper,[b], # Simran Kumari,[a] Nick Heiming,[b] Sonja D. Mürtz,[b] Stephan N. Steinmann,[c] Stefan Palkovits,[b] Regina Palkovits,[b,d], * and Philippe Sautet[a,e], *

[a] Department of Chemical and Biomolecular Engineering,
University of California Los Angeles,
Los Angeles, CA 90095, USA
[b] Chair of Heterogeneous Catalysis and Technical Chemistry
RWTH Aachen University
Worringerweg 2, 52074 Aachen (Germany)
[c] CNRS, Laboratoire de Chimie UMR 5182, ENS de Lyon, 46 allée d'Italie, Lyon F-69342, France
[d] Institute for Sustainable Hydrogen Economy (INW-2), Forschungszentrum Jülich, Marie-Curie-Str. 5, 52428 Jülich, Germany
[e] Department of Chemistry and Biochemistry,
University of California Los Angeles,
Los Angeles, CA 90095, USA

*palkovits@itmc.rwth-aachen.de
*sautet@ucla.edu

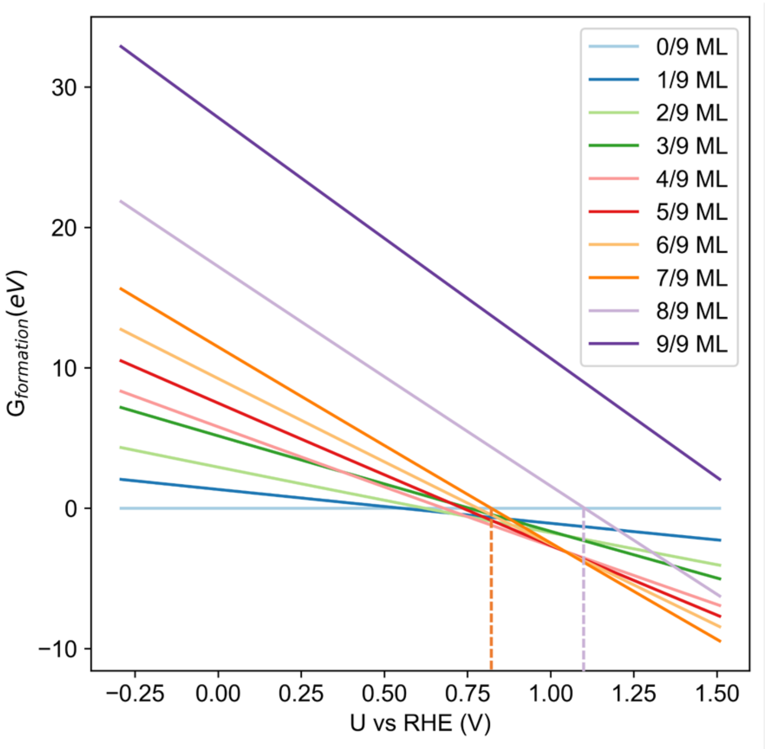

Figure S1. Calculated OH^- adsorption Gibbs free energy on the Cu (111) surface as a function of applied potential. Onset of adsorption occurs at potentials more positive than 0.5 V vs RHE.

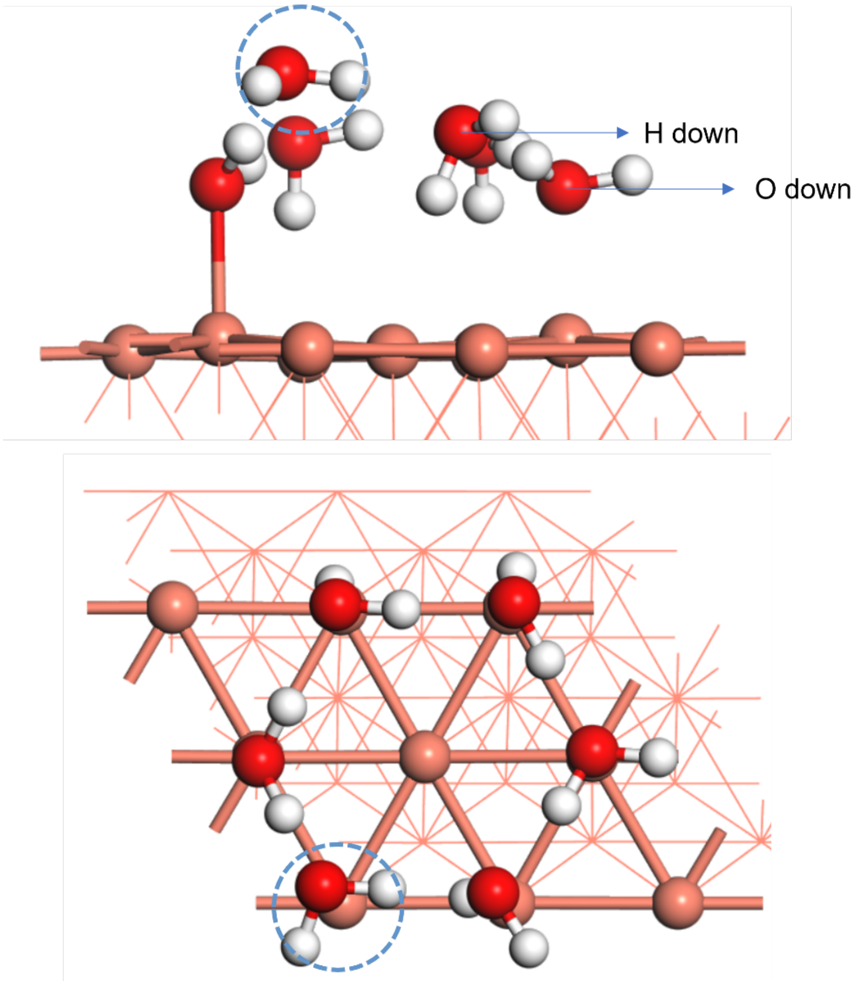

Figure S2. The distribution of H-down and O-down water molecules within one water layer

Chemisorbed molecules are characterized by binding interactions, indicated by short Cu-O distances.

O-down: The oxygen atom is the lowest atom along the surface normal

H-down: One hydrogen atom is the lowest atom along the surface normal points towards the surface, promoting hydrogen bonding and other interactions.

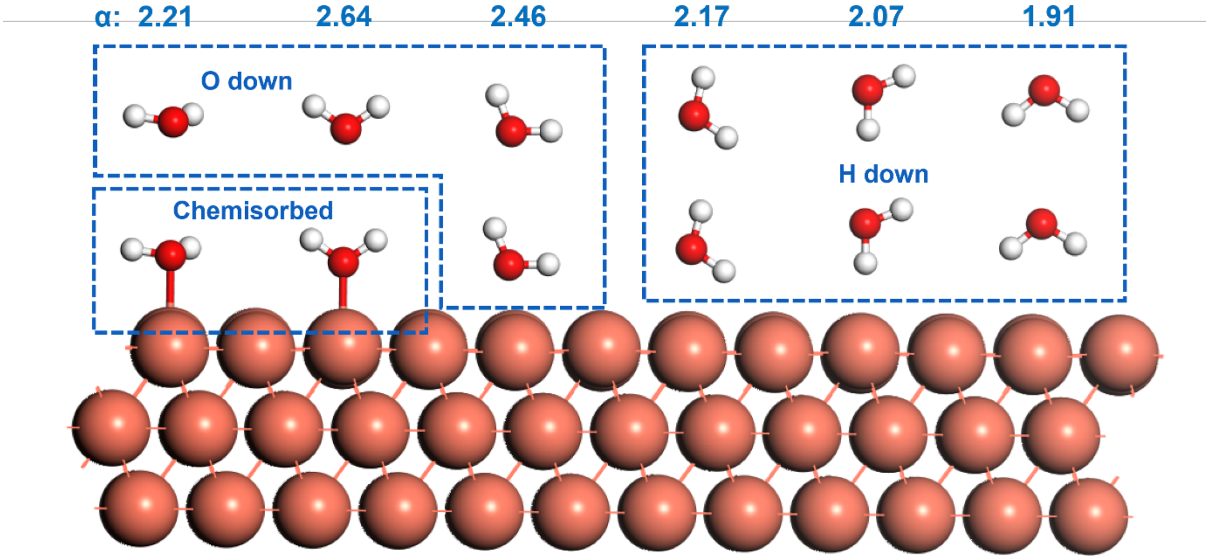

Figure S3. Classification of water molecules' orientations obtained from AIMD sampling, three distinct categories based on the interaction strength and orientation of the molecules on the surface, including chemisorbed, O-down and H-down. Chemisorbed molecules are also O-down molecules.

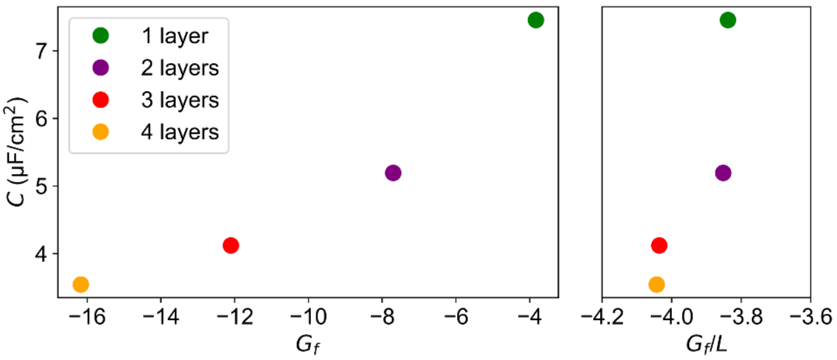

Figure S4. The capacitance and corresponding Gibbs formation energy of one, two, three, and four water layers.

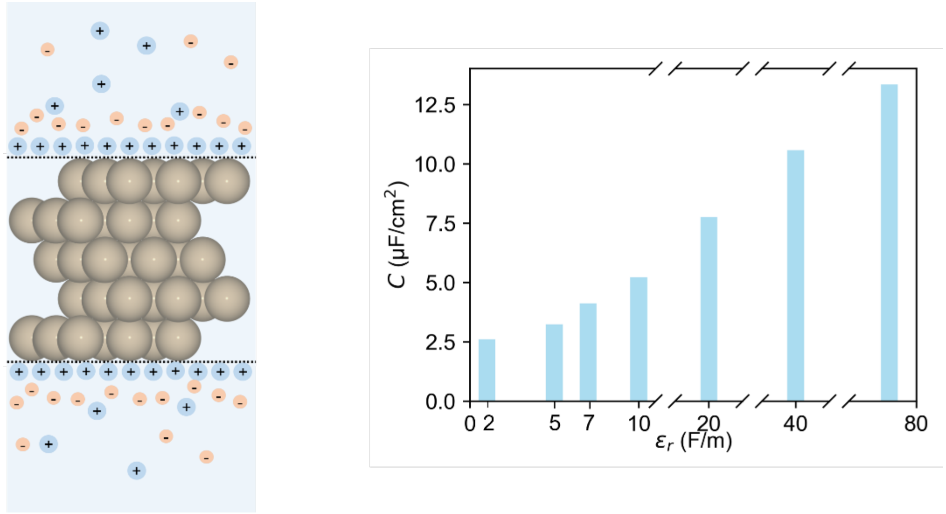

Figure S5. The bare Cu(111) under implicit solvation model of dielectric constant of ε. The relationship between EDL capacitance and dielectric constant is shown on the right

Based on the AIMD sampling, we analyzed the variations in the center of mass and the distribution of water molecule orientations. The results indicate that the movement

of water layers is restricted on the electrode surface. To simplify the classification, **chemisorbed water molecules** are grouped under the **O-down** category, allowing us to focus on two key orientation distributions.

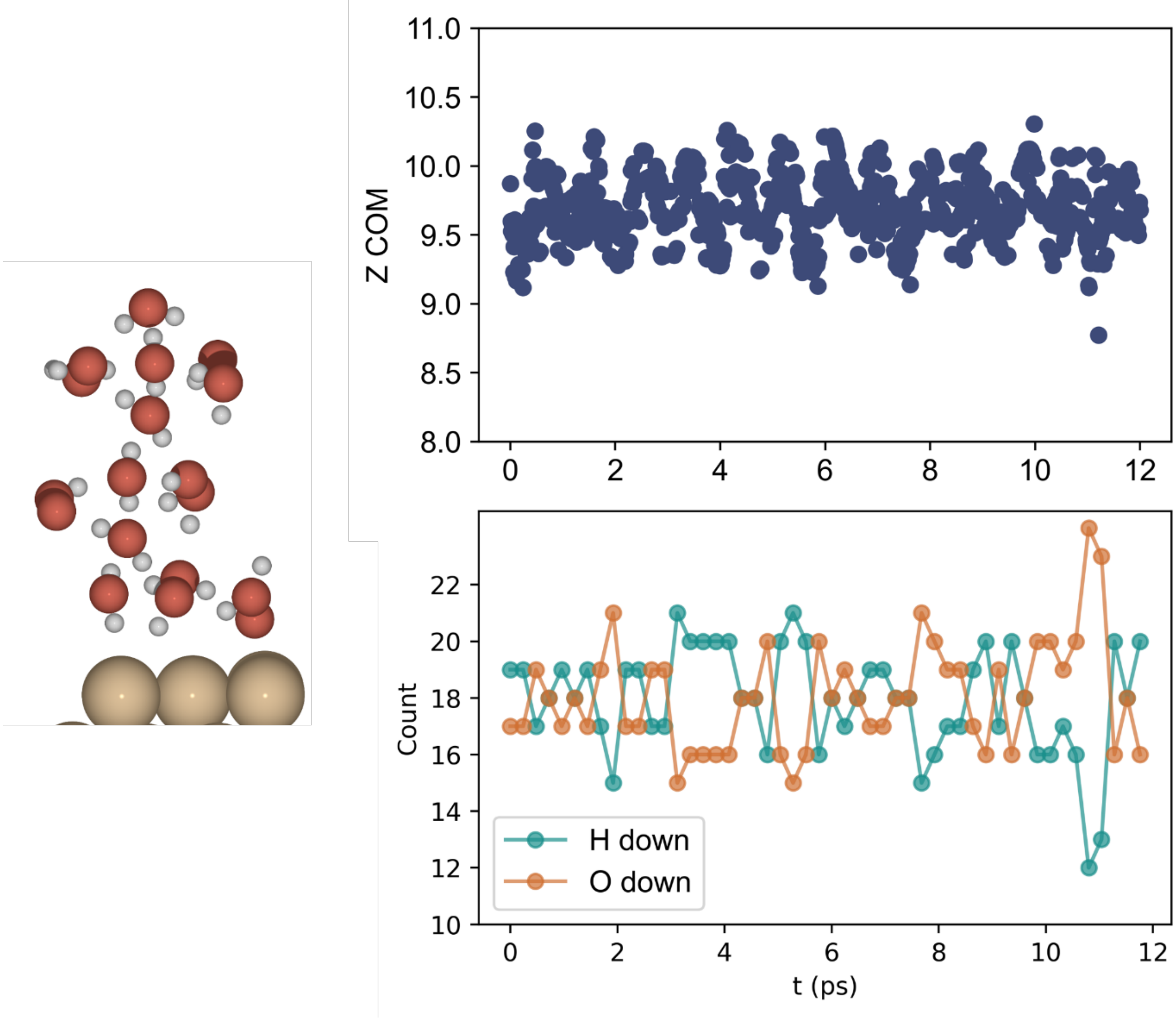

Figure S6. The mass center of water molecules and the distribution of H-down and O-down water molecules within the three water layers model

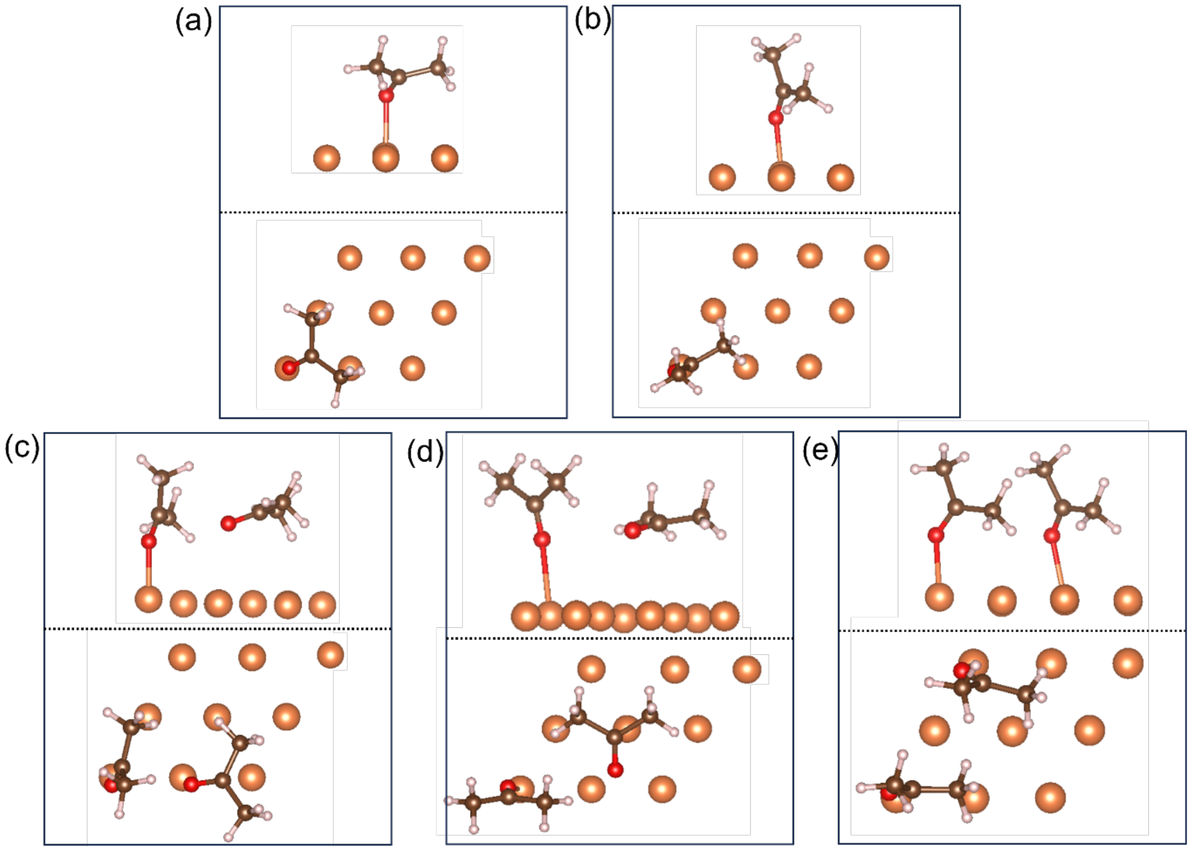

Figure S7. Adsorption modes of acetone on 3x3 Cu(111) surface. (a) and (b) are parallel and vertical modes respectively; (c), (d), and (e) consider the monolayer situations, where two acetones are used

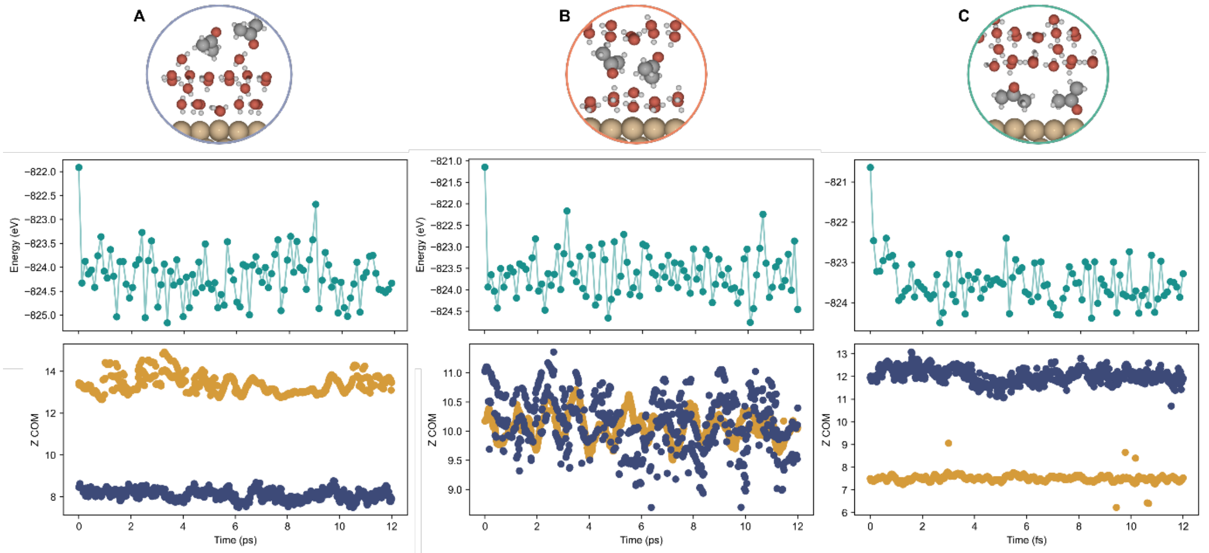

Figure S8. Mass center changes of acetone and water layers in each model. Where dark blue represents water layers and yellow describes acetone

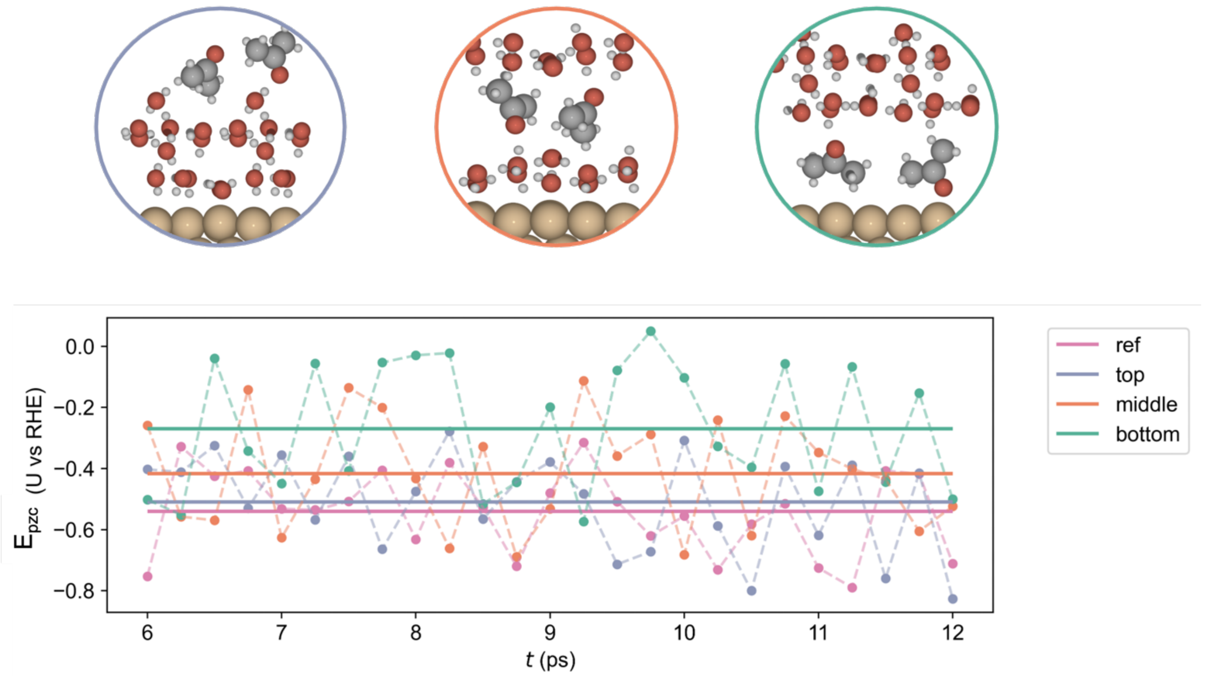

Figure S9. Variation of E_{pzc} for three-layer acetone models along the simulation time

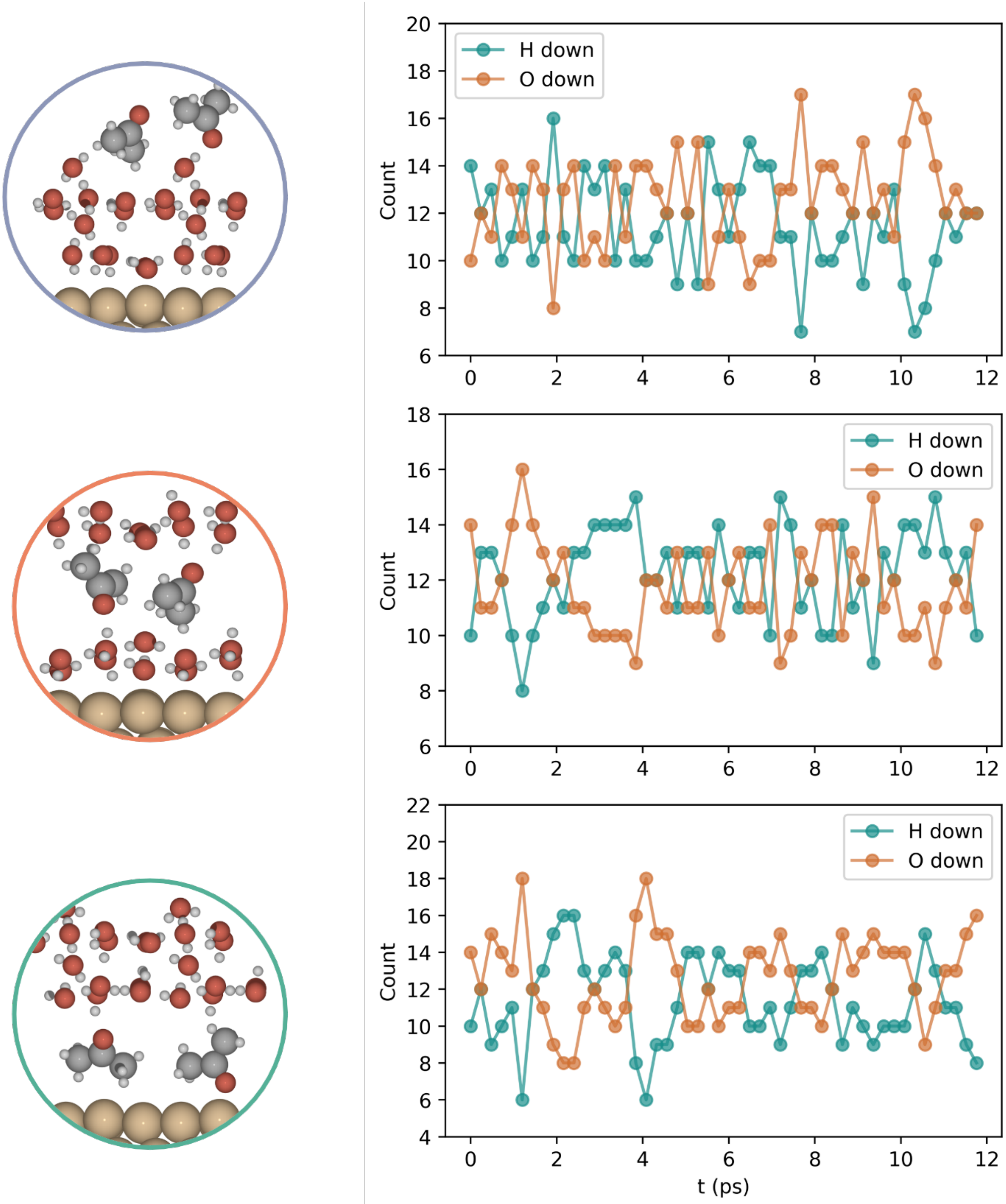

Figure S10. The distribution of H-down and O-down water molecules for each acetone three-layer model

The local potential analysis for three-layer models of acetone at natural conditions, whereas detailed in the Methods Section 4.1 symmetrized models with the box thickness in the *Z* direction of 60 Å were used. As shown the Figure S11, the local potential with distance in z direction has left-right symmetry and the potential reaches a flat, constant value at the boundaries, both on the left (far from the surface) and the right (far from the adsorbed molecules). This flat region in the potential confirms that there are no residual interactions between the repeated images of the periodic cells, as the potential does not exhibit any fluctuations or gradients near the boundaries.

The lack of interaction between the repeated images of the periodic cells is critical for ensuring accurate electrostatic calculations, and these results confirm that the simulation box is appropriately sized, with the cutoff at the boundaries effectively eliminating any spurious long-range interactions.

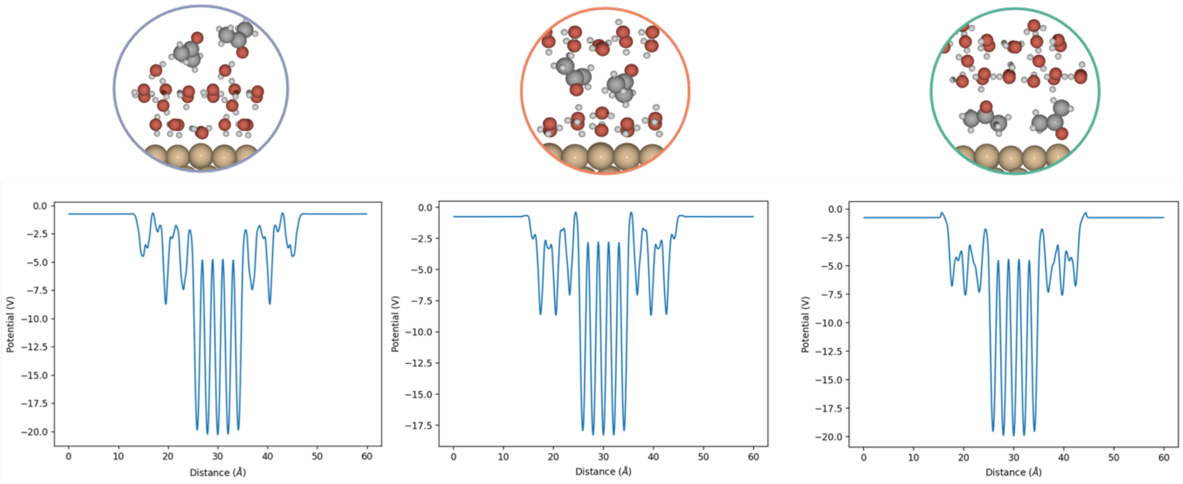

Figure S11. The local potential along z direction for acetone models, acetone in the top, middle and bottom layers from the left to right.

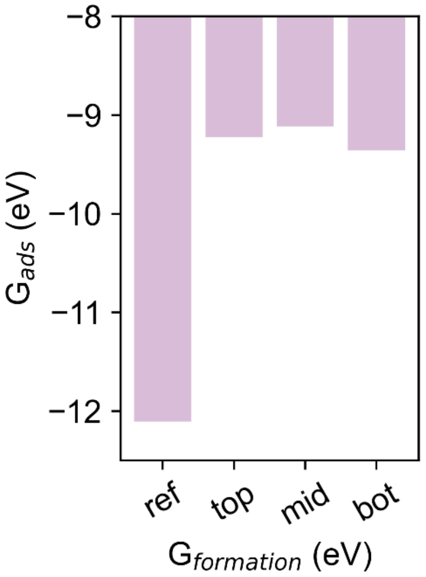

Figure S12. The formation energy for each acetone three-layer model from the last snapshot of AIMD simulations

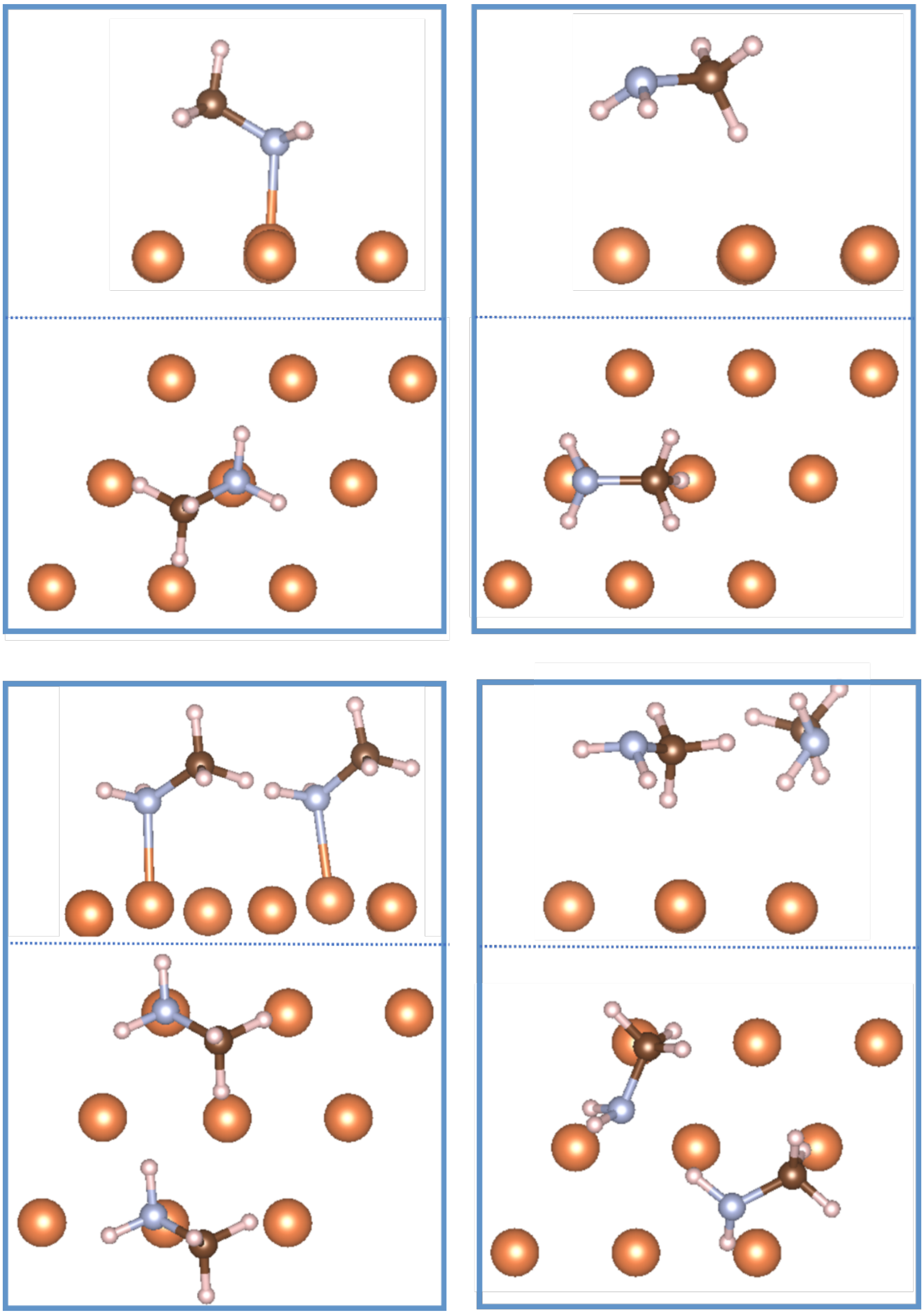

Figure S13. The adsorption modes of methylamine on 3x3 Cu(111) surface, where above two represent one methylamine in vertical and parallel modes, while below two describe two methylamine molecules' adsorption modes

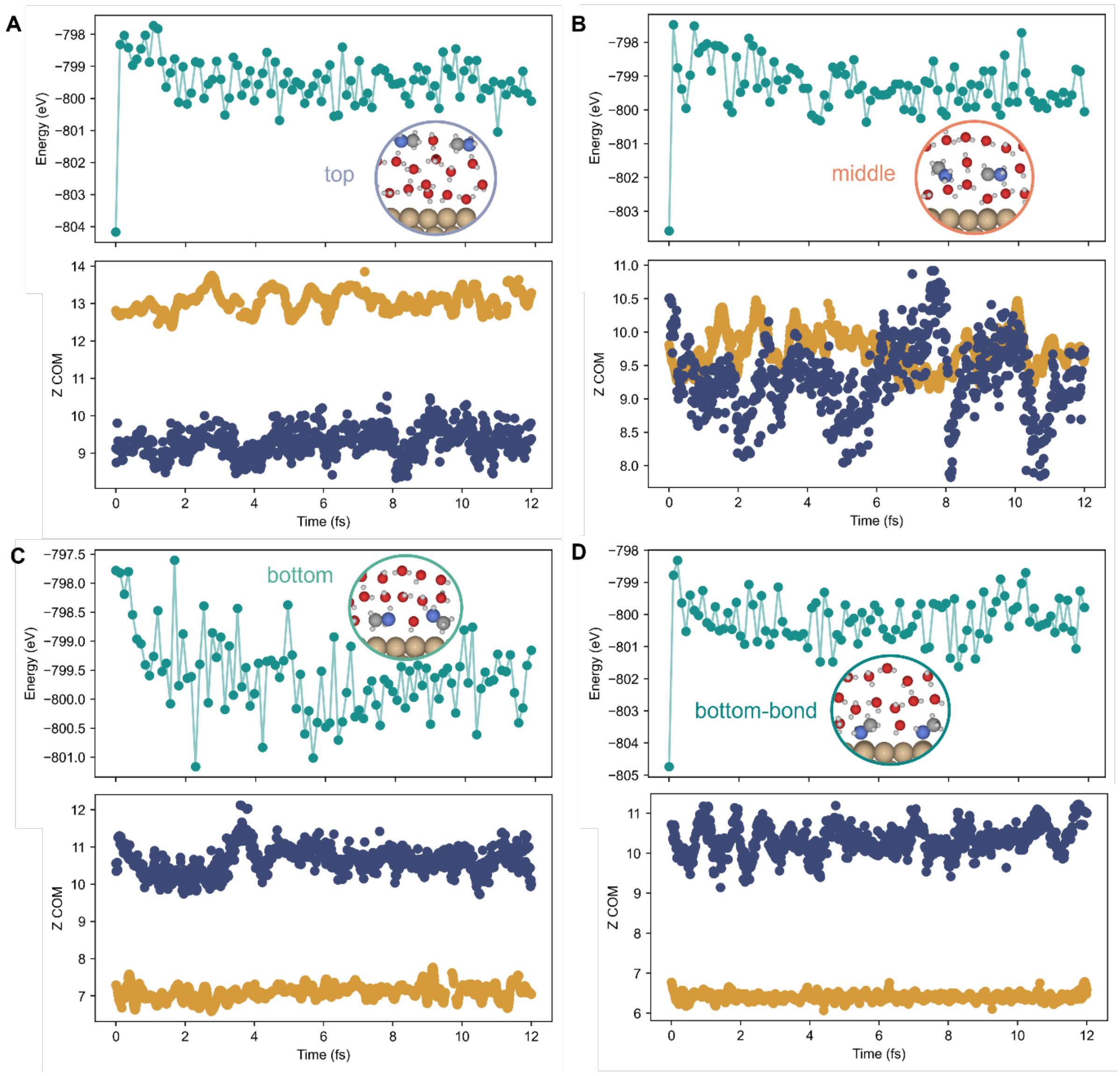

Figure S14. Mass center changes of methylamine and water layers in each model. Where dark blue represents water layers and yellow describes methylamine

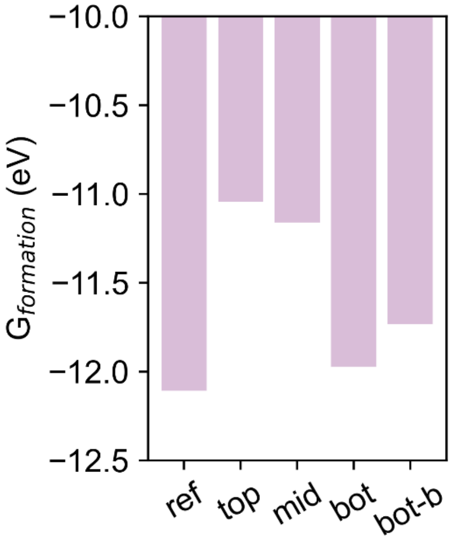

Figure S15. The formation energy for each methylamine three-layer model from the last snapshot of AIMD simulations

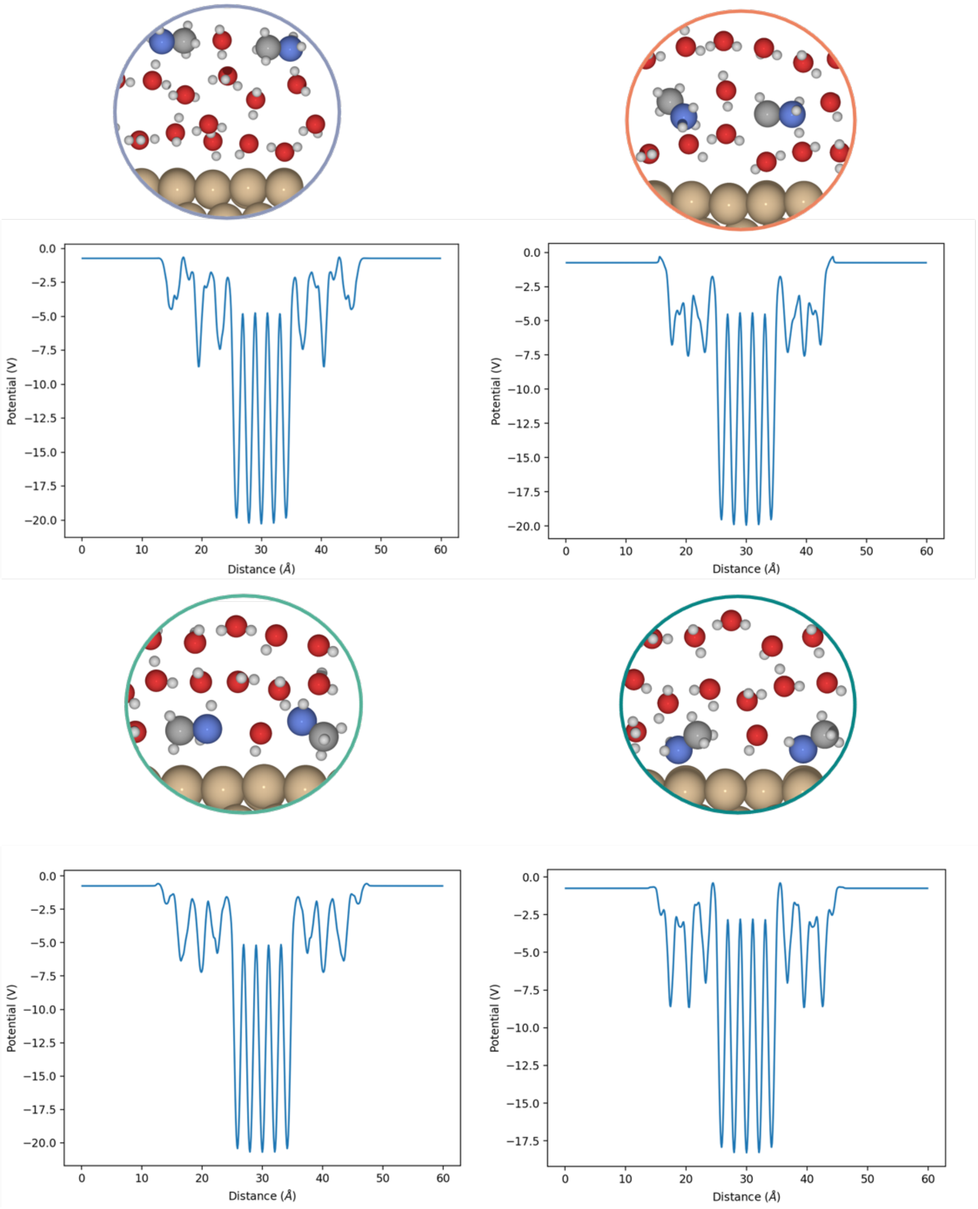

Figure S16. The local potential along z direction for methylamine models, methylamine in the top, middle, bottom and bonded-bottom layers from the left to right.

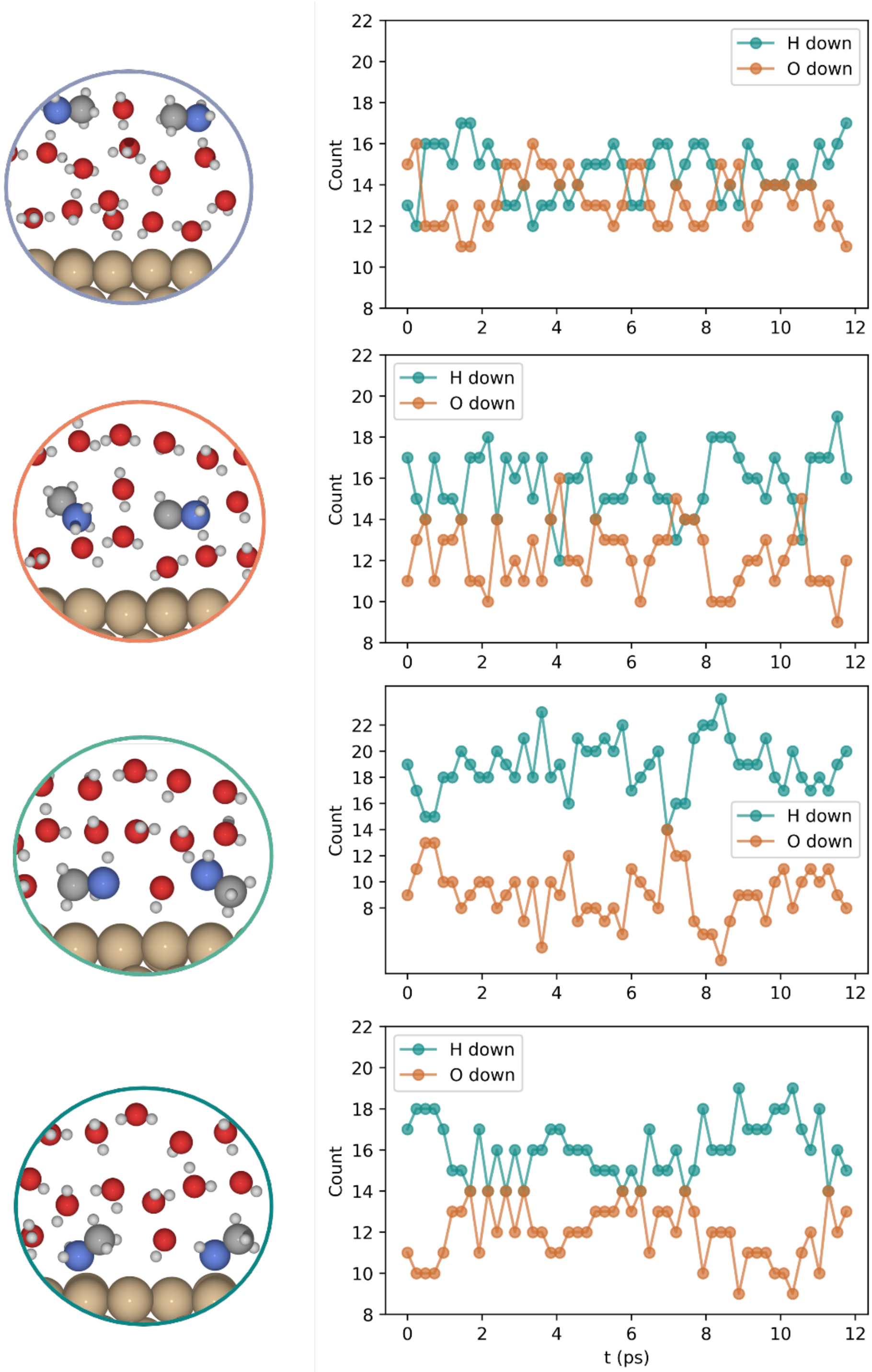

Figure S17. The distribution of H-down and O-down water molecules for each methylamine three-layer model

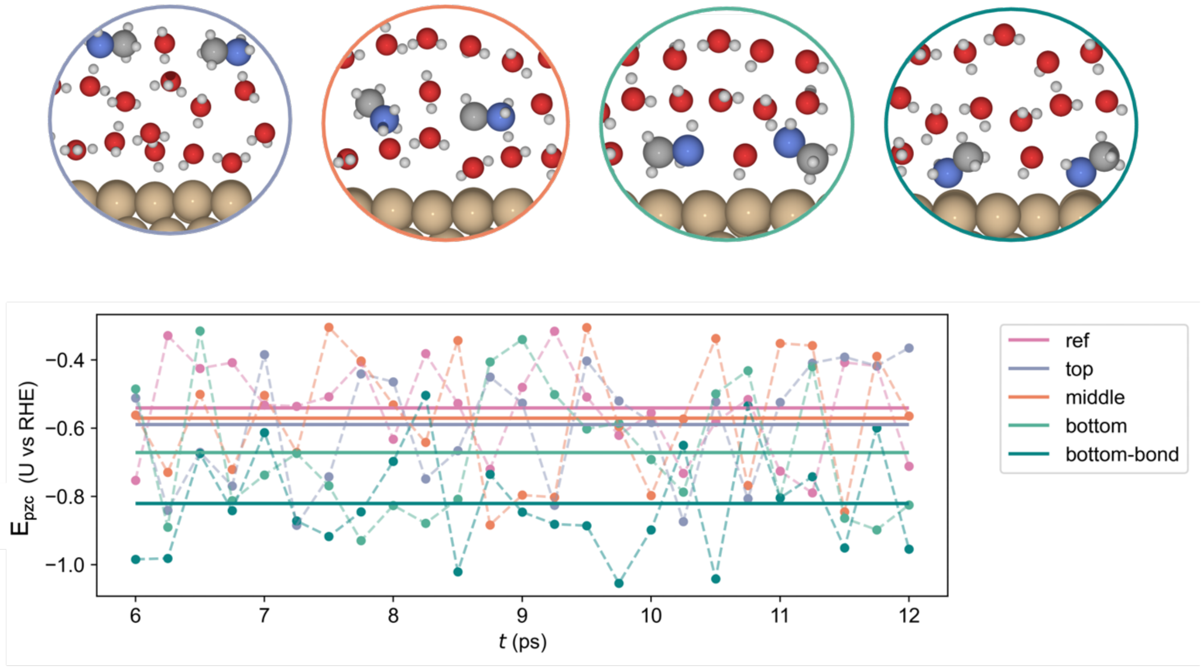

Figure S18. Variation of E_{pzc} for three-layer methylamine models along the simulation time

Table S1. Average value and standard deviation along the trajectory for the potential of zero charge E_{pzc} during the AIMD simulation

	Average E_{pzc} (V vs RHE)	Standard Deviation (V)
Three water layers	-0.54	0.12
Acetone in the bottom layer	-0.27	0.21
Methylamine in the bottom layer	-0.67	0.19
Methylamine in the bottom layer – bonded to surface	-0.82	0.16

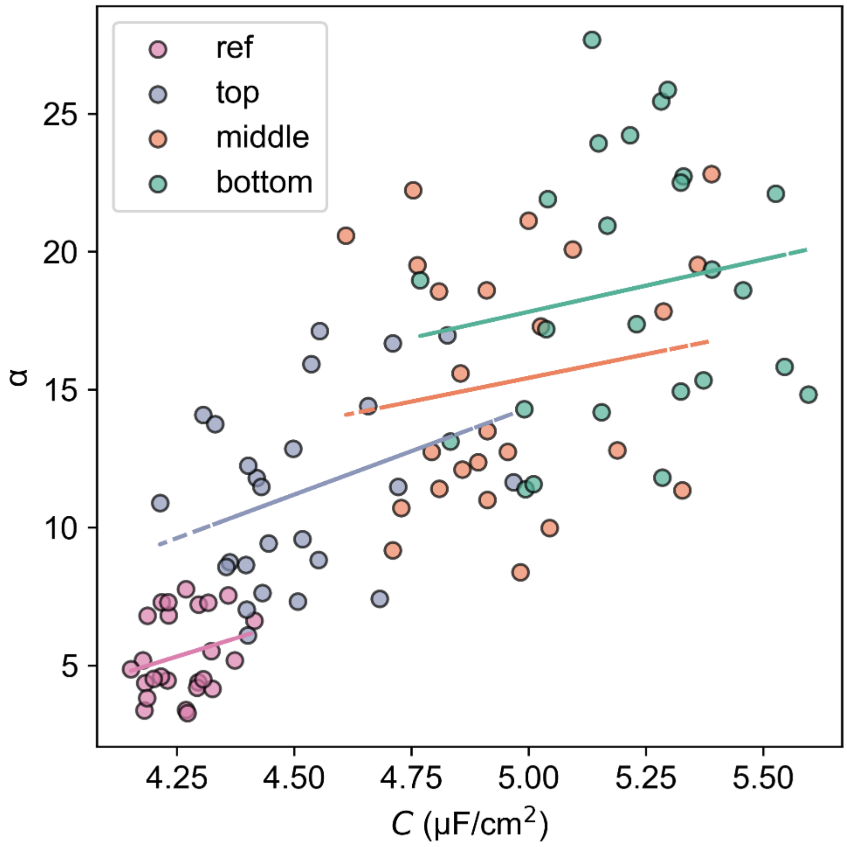

Figure S19. Parity plots (polarizability as a function of capacitance) for acetone models.

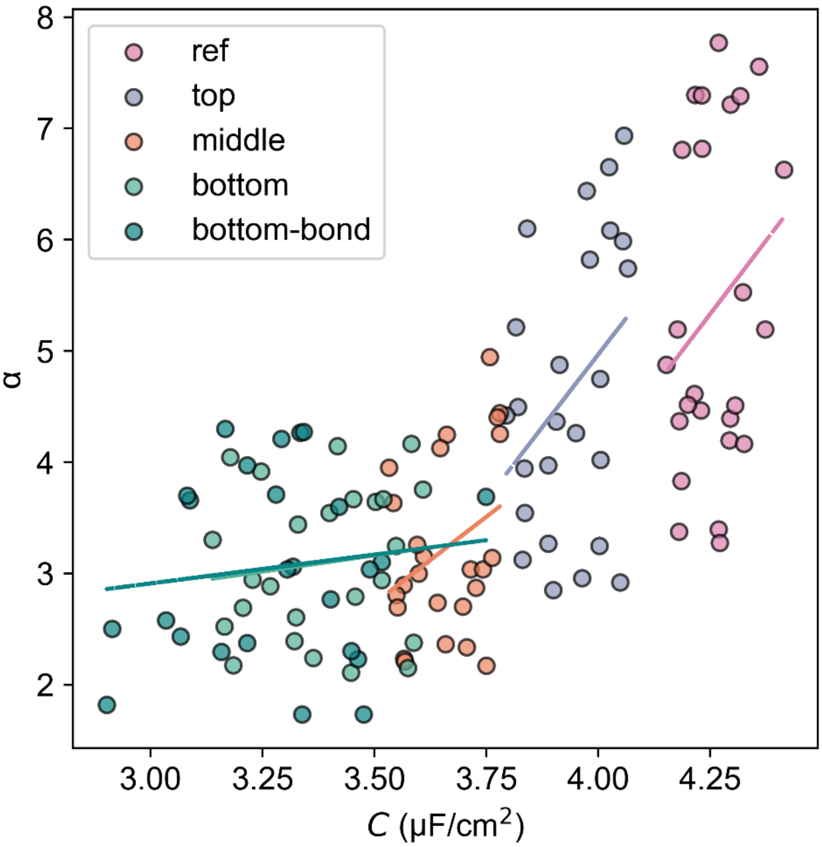

Figure S20. Parity plots (polarizability as a function of capacitance) for methylamine models.

Table S2. Average and RMSD (Root Mean Square Deviation) of water orientations during AIMD simulation

	Ratio of O down	Total water molecules
Three water layers	50.17%	36
Acetone in the top layer	52.25%	24
Acetone in the middle layer	50.25%	24
Acetone in the bottom layer	52.58%	24
Methylamine in the top layer	50.28%	28
Methylamine in the middle layer	43.57%	28
Methylamine in the bottom layer	32.50%	28
Methylamine in the bottom layer-bonded to surface	42.57%	28
